# Diarylheptanoid analogues from the rhizomes of *Zingiber officinale* and their anti-tumour activity†

**DOI:** 10.1039/d1ra03592d

**Published:** 2021-09-02

**Authors:** Ting Li, Da-bo Pan, Qian-qian Pang, Mi Zhou, Xiao-jun Yao, Xin-sheng Yao, Hai-bo Li, Yang Yu

**Affiliations:** Institute of Traditional Chinese Medicine & Natural Products, Guangdong Province Key Laboratory of Pharmacodynamic Constituents of TCM and New Drugs Research, College of Pharmacy, Jinan University Guangzhou 510632 P. R. China 1018yuyang@163.com; Department of Medical Technology, Qiandongnan Vocational & Technical College for Nationalities Kaili Guizhou 556000 P. R. China; Kanion Pharamaceutical Co. Ltd, State Key Laboratory of New-tech for Chinese Medicine Pharamaceutical Process Lianyungang 222001 People's Republic of China lihaibo1985124@sina.com; State Key Laboratory of Applied Organic Chemistry, Department of Chemistry, Lanzhou University Lanzhou 730000 P. R. China

## Abstract

Eight previously undescribed diarylheptanoids (1–8), together with fifteen known analogues (9–23), were isolated from the rhizomes of *Zingiber officinale*. Their structures were unambiguously determined by comprehensive spectroscopic analyses and electronic circular dichroism (ECD) calculations. It is worth mentioning that 1–3 are the first reported structures of diaryl ether heptanoids in *Z. officinale*, whereas 15–17 were isolated from *Zingiber* for the first time. Furthermore, a gene enrichment analysis of the interacting targets indicated that diarylheptanoids were mainly associated with the anti-tumor activity by affecting DNA damage signaling pathway. The results showed that 6, 16–19 had remarkable inhibitory effects against five tumor cell lines (A549, HepG2, HeLa, MDA-MB-231, and HCT116) with IC_50_ values ranging from 6.69–33.46 μM, and showed down-regulating the expression of ATR (ataxia telangiectasia mutated and RAD3-related) and CHK1 (checkpoint kinase 1) levels in HCT116 and A549 cell lines. Our studies not only enrich the structural diversity of diarylheptanoids in nature, but also discover several natural compounds for anti-tumor agents.

## Abstract 2

Diarylheptanoid analogues from the rhizomes of *Zingiber officinale* and their anti-tumour activity.

## Graphical abstract

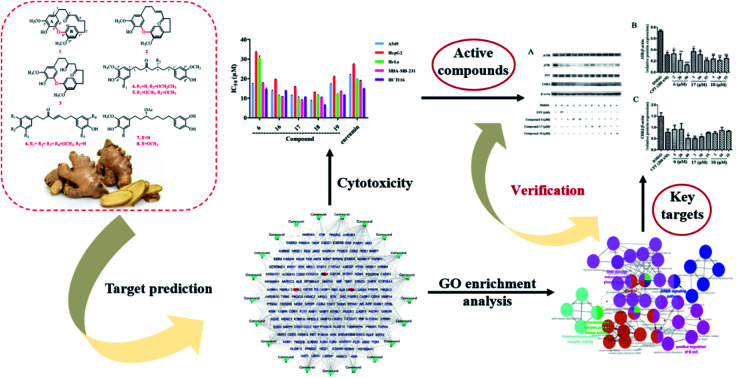

## 1. Introduction

Ginger (*Zingiber officinale* Roscoe; family: Zingiberaceae) has long been used worldwide as an important cooking spice, condiment, and dietary supplement.^1,2^ It is also valued as a medicinal herb for its prevention and treatment of a wide range of diseases, such as colds, stomachaches, headaches, nausea, diarrhoea, indigestion, rheumatic, cardiopathy and cholera.^3–7^ Gingerol-related compounds as well as diarylheptanoids are the major classes of biologically active natural products in ginger. In the past decades, the extensive investigations have been made on ginger and gingerols derivatives.^8,9^ Diarylheptanoids, known as the other significant phenolic components in ginger, have recently attracted widespread attentions for their structural diversity and potential pharmacological effects.

Diarylheptanoids are structurally diverse and have the skeletal structure of two aromatic rings conjugated with seven carbon chains, and is categorized into linear, cyclic, and dimeric diarylheptanoids, or diarylheptanoids with special moieties. Numerous pharmaceutical research revealed that diarylheptanoids possess anti-inflammatory,^10^ anti-oxidant,^11^ anti-tumor,^12^ leishmanicidal,^13^ melanogenesis,^14^ hepatoprotective,^15^ and neuroprotective activities.^16^ A variety of diarylheptanoids have been isolated from seeds, fruits, leaves and roots of plants of different families.^17^ Ginger is one of the most important producers of diarylheptanoids in nature. In our previous study, gingerol derivatives including six new compounds were reported from ginger rhizomes.^18^ As a part of a continuing search for new bioactive agents,^21^ diarylheptanoids including eight undescribed ones (1–8), were identified from the ginger. To rapidly reveal the biological activity, a network pharmacology-based approach was constructed to guide the discovery of targets and biological functions of diarylheptanoid compounds. The results demonstrated that the isolated diarylheptanoids were mainly associated with the antitumor activity by affecting DNA damage signalling pathway. Consequently, cytotoxicity and enzyme activity assays were designed to validate the predictions, suggesting that compounds 6, 17, and 18 may exert anticancer effects by regulating the ATR/CHK1 signalling pathway. Herein, the isolation, structural elucidation, targets prediction, cytotoxic evaluation, enzymatic activity assays, and molecular simulation of these compounds are discussed.

## 2. Materials and methods

### 2.1. Instrumentation and reagents

Ultraviolet spectra (UV) were recorded on a JASCO V-550 UV spectrometer (JASCO, Tokyo, Japan). Optical rotations were determined in CHCl_3_ using a JASCO P-1020 polarimeter (JASCO, Tokyo, Japan). Circular dichroism (CD) spectra were tested by JASCO J-810 circular dichroism spectrometer (Jasco, Tokyo, Japan). Infrared spectra (IR) were measured with a JASCO FT/IR-480 plus spectrometer (JASCO, Tokyo, Japan). 1D and 2D NMR spectra were acquired on a Bruker AV 600 (Bruker Co. Ltd, Bremen, German) using solvent signals (CDCl_3_: *δ*_H_ 7.26/*δ*_C_ 77.2; CD_3_OD: *δ*_H_ 3.31/*δ*_C_ 49.0) as internal references. Deuterated solvents were purchased from Cambridge Isotope Laboratories, Inc. (Saint Louis, Missouri, USA). The HR-ESI-MS spectra were obtained on a Micromass Q-TOF mass spectrometer (Waters Corporation, Milford, USA). Analytical high-performance liquid chromatography (HPLC) was carried out on a Shimadzu HPLC system (Shimadzu, Kyoto, Japan) with an LC-20AB solvent delivery system and an SPD-20A UV/vis detector using a Phenomenex Gemini C_18_ column (5 μm, *Φ* 4.6 × 250 mm; Phenomenex Inc., Los Angeles, USA). Semi-preparative HPLC was performed on a Shimadzu HPLC system (Shimadzu, Kyoto, Japan) equipped with LC-6AD solvent delivery system and a SPD-20A detector on a Phenomenex Gemini C_18_ column (5 μm, *Φ* 10.0 × 250 mm; Phenomenex Inc., Los Angeles, USA).

Column chromatography (CC) was carried out on silica gel (300–400 mesh, Haiyang Chemical Co. Ltd, Qingdao, China), ODS (12 nm, S-50 μm, YMC Ltd, Tokyo, Japan) and Sephadex LH-20 (Amersham Pharmacia Biotech Co. Ltd, Atlanta, USA). Thin-layer chromatography (TLC) was performed on pre-coated silica gel plates (GF254, Qingdao Haiyang Co. Ltd, Qingdao, China). HPLC-grade methanol and acetonitrile (CH_3_CN) were purchased from Oceanpak Alexative Chemicals Co. Ltd. (Gothenburg, Sweden). All analytical grade reagents were from Concord Chemicals Co. Ltd (Tianjin, China).

### 2.2. Plant materials

Dried rhizomes of *Z. officinale* were collected from Jinxiang County, Shandong, China, in September 2016. The plant material was identified by Prof. Guang-Xiong Zhou of Jinan University. A voucher specimen (JNU-ZO-201609) was deposited at the Institute of Traditional Chinese Medicine and Natural Products, College of Pharmacy, Jinan University, Guangzhou, China.

### 2.3. Extraction and isolation

The dried rhizomes (30 kg) of *Z. officinale* were extracted with 70% ethanol (210 L) under reflux for 2 times at 60 °C, 2 h each time. The combined ethanol extract was evaporated under reduced pressure. The ethanol extract (5.5 kg) was suspended in 95% ethanol (16.5 L) and partitioned successively with petroleum ether and ethyl acetate. The ethyl acetate portion (ZO-2, 920 g) was divided into 22 fractions (Fr. A to Fr. V) on a silica gel column chromatograph (CC) (200–300 mesh, *Φ* 6.5 × 130 cm) by gradient elution with solvent composed of cyclohexane–EtOAc (v/v, 97 : 3, 95 : 5, 9 : 1, 8 : 2, 7 : 3, 6 : 4, and 0 : 1).

Fr. O (22 g) was eluted (CH_3_OH–H_2_O, 5 : 5 → 10 : 0) by ODS to yield 11 fractions (Fr. O1–O11). Fr. O7 was separated by silica gel CC (CHCl_3_–acetone 95 : 5, v/v) to give 4 fractions (Fr. O7A–O7D). Compounds 1 (1.4 mg, *t*_R_ = 35.7 min), and 2 (1.0 mg, *t*_R_ = 36.5 min) were obtained from Fr. O7A by semi-preparative HPLC (45% CH_3_OH). Subsequently, Fr. O9 was purified by preparative HPLC to yield compound 4 (14.1 mg, *t*_R_ = 84 min, 53% CH_3_OH).

Fr. Q (16.0 g) was subjected to ODS eluting with CH_3_OH–H_2_O (5 : 5 → 10 : 0) to afford Fr. Q1–Q17. Among them, compound 7 (2.9 mg, *t*_R_ = 37.5 min, 58% CH_3_CN) was purified by semi-preparative HPLC from Fr. Q10. Fr. Q7 was separated by semi-preparative HPLC (29% CH_3_CN) to yield 9 fractions (Fr. Q7A–Q7I), Fr. Q7I and Q7M were further treated by semi-preparative HPLC to obtain compounds 5 (1.3 mg, *t*_R_ = 18.2 min, 50–60% CH_3_OH, 40 min) and 6 (3.0 mg, *t*_R_ = 19.5 min, 50–70% CH_3_OH, 40 min), respectively.

Fr. R (29.0 g) was applied to a ODS column using CH_3_OH–H_2_O (5 : 5 → 10 : 0) as elute to obtain 11 fractions (R1–R11). Then, Fr. R4 was treated by semi-preparative HPLC eluting with 23% CH_3_CN to obtain a mixture (R4G), and further purified by semi-preparative HPLC (50% CH_3_OH) to yield compound 3 (4.8 mg, *t*_R_ = 11.9 min). Fr. R9 was treated by semi-preparative HPLC eluting with 42% CH_3_CN, and compound 8 (11.6 mg, *t*_R_ = 25.6 min) was obtained. In addition, detailed isolation procedures for known compounds (9–23) and spectroscopic data of isolated new compounds are provided in the ESI.†

### 2.4. ECD calculation

The ECD spectra of (5*R*)-3 and (5*S*)-3 were calculated using Gaussian 09 software. Firstly, predominant conformations were obtained using conformation searches. Secondly, all conformations were optimized at B3LYP/6-31G(d) level. Thirdly, the B3LYP/6-311+G(d,p) level was used to calculate the compound's ECD spectra. Finally, The ECD spectra were combined after Boltzmann weighting based on the distribution of structural energy.

### 2.5. Compound-target network construction and analysis

The targets of the isolated compounds were predicted by using SwissTargetPrediction,^19^ and the cancer-related targets were extracted from GenCLip3.^20^ Next, these targets were merged to obtain the overlapped targets, and the compounds-antitumor targets network was built by using the overlapped targets and their corresponding compounds. The key anticancer targets were identified based on the intermediate value of degree. Furthermore, the potential biological activity of these isolated-compounds were predicted by using ClueGO program in Cytoscape 3.5 software.^21^

### 2.6. Assays for cell proliferation

#### 2.6.1. Cell culture

Five tumour cell lines A549, HepG2, HeLa, MDA-MB-231, and HCT116 were obtained from ATCC and inoculated in DMEM supplemented with 10% fetal bovine serum (FBS, Gibco, NewYork, USA) under a humidified atmosphere of 95% air and 5% CO_2_ at 37 °C. The culture medium was changed every day. The cultured cell morphology was compared with the normal cell morphology in the ATCC cell bank to determine the cell status.

#### 2.6.2. Cell viability assay

The colorimetric [3-(4,5-dimethyl-thiazol-2-yle) 2,5-diphenyltetrazolium bromide] (MTT, Aladdin, Shanghai, China) assay was employed to quantify the cell proliferation. Five tumour cells were seeded in 96-well plates (100 μL medium per well) at a concentration of 5 × 10^3^ cells per well, and cultured for 24 h. Subsequently, the media were removed and replaced by fresh medium containing different concentrations of drugs, and the control group was incubated with drug-free medium. After culturing for 24 h, the original medium was discarded and replaced by 100 μL complete medium with 0.5 mg mL^−1^ 20 μL MTT. After incubation with 5% CO_2_-95% air at 37 °C for 4 h, the MTT solution was removed and followed by adding 150 μL DMSO, and the plates were shaken for 5 min to dissolve the crystals fully. The optical density of each condition was measured by the microplate reader (BioTek, Vermont, USA) at a wavelength of 490 nm. Each experiment was repeated in triplicates.

#### 2.6.3. Western blotting

The HCT116 cells were seeded in 60 mm dishes overnight and treated with the indicated compounds. Harvested cells were disrupted with cell lysis buffer (50 mM Tris–HCl, pH 8.0; 150 mM NaCl; 0.5% Na-deoxycholate; 1% NP-40; 0.1% SDS), and with protease inhibitor mixture. After sonication, the cell lysates were centrifuged at 14 000*g* and 4 °C for 15 min. Protein concentration of supernatant was determined. Equal amounts of lysate protein were separated by SDS–polyacrylamide gel electrophoresis and electrotransferred to polyvinylidene difluoride (PVDF) membranes. The membranes were blocked with 5% nonfat skim milk in TBST [20 mM Tris–HCl (pH 7.6), 135 mM NaCl and 0.1% Tween 20], and then incubated with specific primary antibodies to ATM, ATR, and P53 (Cell Signalling Technology, Danvers, MA) at 4 °C overnight. β-Actin was used as a loading control. The next day, the membrane was incubated with goat anti-mouse IgG–HRP secondary antibody (Cell Signalling Technology, Danvers, MA) at room temperature for 2 h. The signals were detected by chemiluminescence utilizing the enhanced chemiluminescent reagent and recorded on the gel imaging system (Tanon, Shanghai, China) and relative quantified using Image J software program. The experiment was repeated three times, and A549 cells were tested in the same way. The test results of A549 cell line are provided in the ESI (Fig. S72†).

#### 2.6.4. Statistical analysis

Quantitative data were presented as mean ± SD. The statistical analysis was performed by GraphPad Prism 4.0 (GraphPad Software Inc). The statistical significance between groups was interpreted by one-way ANOVA analysis of variance, followed by Tukey's test. All statistical tests with *p* < 0.05 were considered significantly different.

## 3. Results and discussion

### 3.1. Structural elucidation of new compounds

**Fig. 1:**
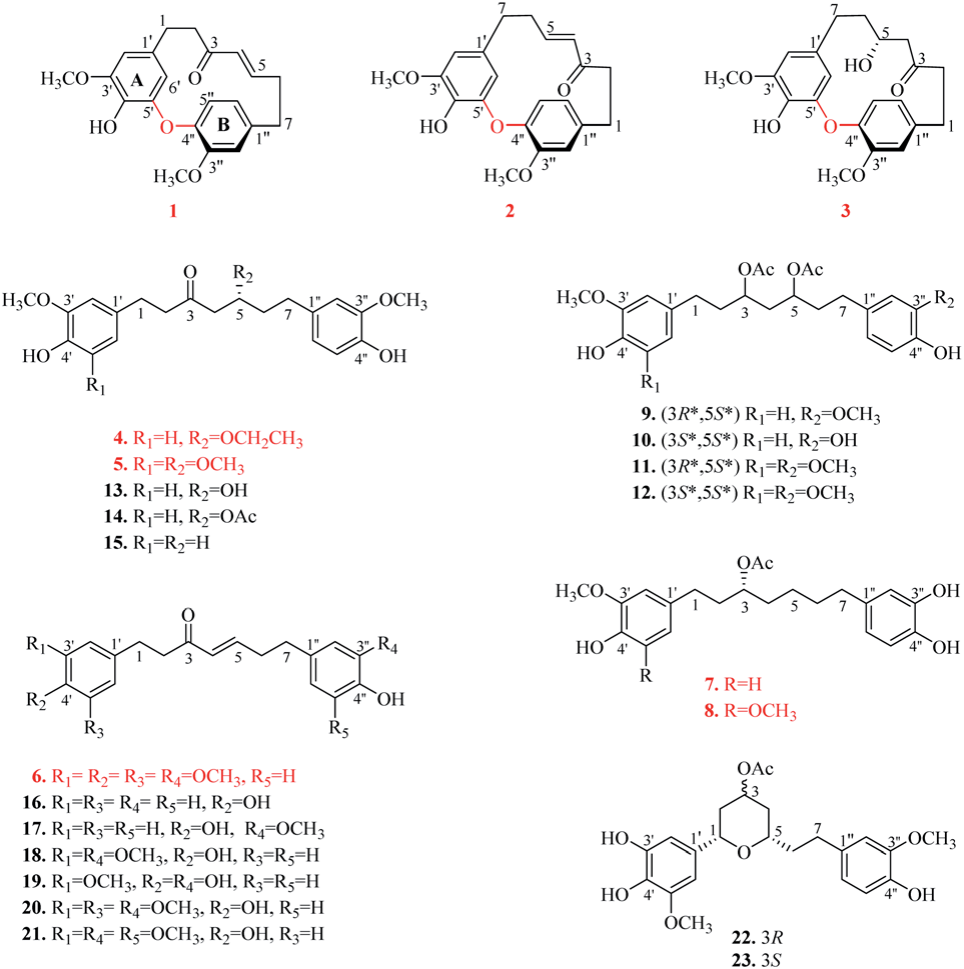
Structures of compounds 1–23.

**Fig. 2:**
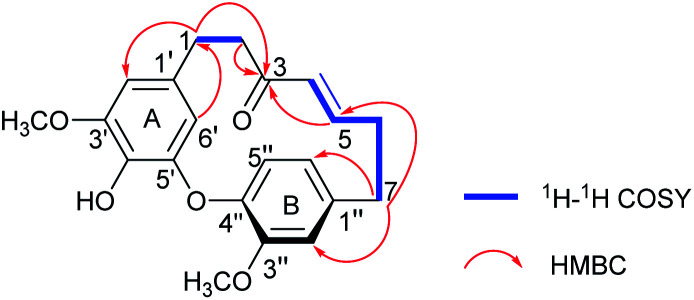
Key ^1^H–^1^H COSY and HMBC correlations of compound 1.

Compound 1 was isolated as a yellow oil. The molecular formula was determined to be C_21_H_22_O_5_ on the basis of the HR-ESI-MS (*m*/*z* 355.1539 [M + H]^+^, calcd 355.1545). The ^1^H NMR spectrum showed diagnostic signals of one 1,2,4-trisubstituted benzene [*δ*_H_ 6.92 (1H, d, *J* = 8.0 Hz, H-5′′), 6.80 (1H, d, *J* = 1.8 Hz, H-2′′), and 6.73 (1H, dd, *J* = 8.0, 1.8 Hz, H-6′′)] and one 1,2,3,5-tetrasubstituted benzene [*δ*_H_ 6.38 (1H, d, *J* = 1.8 Hz, H-2′), and 4.99 (1H, d, *J* = 1.8 Hz, H-6′)], a *trans*-substituted double bond [*δ*_H_ 6.58 (1H, ddd, *J* = 15.2, 8.6, 7.5 Hz, H-5), 5.86 (1H, d, *J* = 15.2 Hz, H-4)], and two methoxyls [*δ*_H_ 3.82 (3H, s, 3′-OCH_3_), 3.68 (3H, s, 3′′-OCH_3_)]. The ^13^C NMR and DEPT data revealed the presence of 21 carbon signals (Table 1), including eight quaternary carbons (*δ*_C_ 201.1, 154.0, 149.9, 149.6, 144.1, 140.8, 134.1, 132.6), seven methines (*δ*_C_ 147.9, 131.8, 125.1, 122.5, 115.8, 106.5, 106.3), four methylenes, and two methoxy carbon signals. The above NMR data supported 1 to be a cyclic diarylheptanoid.^22^ The structural elucidation of 1 was accomplished by analysis of COSY, HSQC and HMBC data (Fig. 2). In particular, the ^1^H–^1^H COSY correlations of H_2_-1/H_2_-2 and H-4/H-5/H_2_-6/H_2_-7, combined with the HMBC correlations of H_2_-1, H_2_-2/C-3, H-4, H-5/C-3, and H_2_-7/C-5 revealed the presence of a heptane chain moiety. Further, two *meta*-coupling doublets, associated with chemical shift characteristics, and key HMBC peaks allowed the assignments of aromatic ring A. In aromatic B, HMBC correlations from a broad *ortho* doublet H-5′′ to C-1′′, 3′′, 4′′, from a narrow *meta* doublet H-2′′ to C-3′′, 4′′, 1′′, 6′′, and from a doublet of doublets to C-1′′, 2′′, 4′′, completed its assignments of the signals. The linkage of alkyl and aromatic groups was built by the HMBC correlations of H_2_-7 to C-1′′, 2′′, 6′′, H_2_-2′′, 6′′ to C-7, as well as H-2′, 6′/C-1, and H_2_-1/C-1′, 2′, 6′, indicating the connection of C-1/C-1′and C-7/C-1′′. The HMBC correlations between *δ*_H_ 3.82 (3′-OCH_3_) with *δ*_C_ 149.6(C-3′) and *δ*_H_ 3.68 (3′′-OCH_3_) with *δ*_C_ 154.0 (C-3′′), demonstrated that the methoxy groups were located at C-3′ and C-3′′, respectively. Moreover, the NMR data the H-6′ (*δ*_H_ 4.99) signal appeared abnormally up-field from other aromatic proton signals, which is the distinguished characteristic of diphenyl ether type cyclic diarylheptanoid. Combined with HR-ESI-MS data, it is confirmed that 1 is diphenyl ether type cyclic diarylheptanoid.^23^ Furthermore, 1 showed positive optical rotation ([*α*]^25^_D_ +22.7), Since 1 has a chiral plane in the molecule.^24–26^ Thus, 1 was elucidated as shown in Fig. 1 and named (+)-cyclogingerenone A. Unfortunately, the absolute stereochemistry remained undetermined.

**Table 1:** ^1^H (600 MHz) and ^13^C (150 MHz) NMR data of compounds 1–3

Pos.	1^a^		2^b^		3^b^	
	*δ*_C_	*δ*_H_ (*J* in Hz)	*δ*_C_	*δ*_H_ (*J* in Hz)	*δ*_C_	*δ*_H_ (*J* in Hz)
1	29.2	2.85, ddd (16.4, 8.7, 2.4)	33.6	3.01	32.9	2.96, ddd (12.9, 5.8, 2.9)
		2.95				3.04, dt (12.9, 5.8)
2	42.0	2.49, ddd (15.0, 8.7, 2.4)	43.9	2.64	44.6	2.51
						2.79, dt (12.2, 5.8)
3	201.1		200.8		215.6	
4	131.8	5.86, d (15.2)	131.4	5.73, dt (15.3, 1.5)	52.4	1.89, d (18.3)
						2.22, dd (18.3, 10.5)
5	147.9	6.58, ddd (15.2, 8.6, 7.5)	148.2	6.56, dt (15.3, 7.0)	66.1	3.34, br t (8.4)
6	35.1	2.44	32.8	2.34	34.8	1.65
		2.59, dq (12.6, 6.3)				1.33
7	34.6	2.95	32.0	2.70	29.0	2.49
						2.72, ddd (16.8, 11.4, 2.1)
1′	132.6		132.7		131.4	
2′	106.5	6.38, d (1.8)	105.7	6.28, d (1.7)	105.9	6.34, br s
3′	149.6		147.3		147.9	
4′	134.1		132.9		133.1	
5′	149.9		147.9		147.6	
6′	106.3	4.99, d (1.8)	106.8	5.52, d (1.7)	107.4	5.33, br s
1′′	140.8		138.5		138.0	
2′′	115.8	6.80, d (1.8)	114.9	6.77, d (1.8)	116.2	6.59, d (1.8)
3′′	154.0		152.7		151.9	
4′′	144.1		143.5		145.2	
5′′	125.1	6.92, d (8.0)	124.6	7.01, d (8.0)	124.7	7.21, d (8.0)
6′′	122.5	6.73, dd (8.0, 1.8)	121.8	6.83, dd (8.0, 1.8)	120.2	6.93, dd (8.0, 1.8)
3′-OCH_3_	56.7	3.82, s	56.4	3.87, s	56.4	3.87, s
3′′-OCH_3_	56.6	3.68, s	56.5	3.72, s	56.3	3.59, s

a Measured in CD_3_OD.

b Measured in CDCl_3_.

Compound 2 was separated as a yellow oil. The molecular formula of 2 was deduced as C_21_H_22_O_5_ according to HR-ESI-MS ion peak at *m*/*z* 355.1553 [M + H]^+^ (calcd for C_21_H_23_O_5_, 355.1545). Comprehensive analysis of the NMR data (see Table 1) demonstrated that the structure of 2 was quite similar to 1, and the difference between them was the connection sites of the benzenes and heptane moiety. The HMBC correlations from H_2_-1 to C-3, 2′′, 6′′, H_2_-7 to C-2′, 6′, 5, and H-4, H-5/C-3, 7, inferred that C-1 and C-7 were connected to C-1′′ and C-1′, respectively. In addition, 2 showed negative optical rotation ([*α*]^25^_D_ −17.5). Therefore, 2 was elucidated as a new diaryl ether heptanoid and named (−)-cyclogingerenone B.

The molecular formula of compound 3 was determined as C_21_H_24_O_6_ by HR-ESI-MS (*m*/*z* 373.1654 [M + H]^+^; calcd 373.1651). It was disclosed that the only structural difference between 3 and 2 was concerned the change of the heptane chain moiety. The NMR data of 3 revealed the presence of one more oxymethine [*δ*_H_ 3.34 (1H, br t, *J* = 8.4 Hz, H-5); *δ*_C_ 66.1 (C-5)] and one more methylene [*δ*_H_ 1.89 (1H, d, *J* = 18.3 Hz, H-4a), 2.22 (1H, dd, *J* = 18.3, 10.5 Hz, H-4b); *δ*_C_ 52.4 (C-4)], but one less *trans*-double bond by comparing with 2. Consequently, the detailed analysis of the spectroscopic data allowed the planar structure of 3.

The absolute configuration of C-5 in 3 was established by comparison of the calculated ECD spectra [(5*R*)-3 and (5*S*)-3] with the experimental counterpart (see ESI†). The calculated ECD spectrum (Fig. S70 and 71†) (5*R*)-3 showed good consistency with the experimental one. In addition, 3 showed positive optical rotation ([*α*]^25^_D_ +2.7). Thus, the structure of 3 was assigned as (+)-cyclogingerenone C.

Compound 4 was obtained as a yellow gum. The HR-ESI-MS of 4 showed a quasimolecular peak at *m*/*z* 403.2110 [M + H]^+^ (calcd for C_23_H_30_O_6_, 403.2121), corresponding to the molecular formula of C_23_H_30_O_6_. Careful analysis of its ^1^H and ^13^C NMR data (Table 2) indicated that 4 had a similar structure to 13. The main difference found between them concerned the substituent group at C-5. A hydroxyl group at C-5 in 13 was changed to an ethoxy group in 4. This deduction was supported by ^1^H–^1^H COSY correlations of H_2_-8/H_3_-9 and HMBC cross peaks from H-8 [*δ*_H_ 3.42 (2H, qd, *J* = 7.1, 2.0 Hz)] to C-5 (*δ*_C_ 76.3). The absolute configuration of C-5 of 4 was determined by comparing the optical rotation with analogues.^27–30^ The negative optical rotation ([*α*]^25^_D_ −10.7) of 4 indicated 5*R* configuration. On the basis of the above evidence, the structure of 4 was determined (Fig. 1) and named (5*R*)-5-ethoxyhexahydrocurcumin.

**Table 2:** ^1^H and ^13^C NMR data of compounds 4–6

Pos.	4^a^^,^^c^		5^b^^,^^d^		6^b^^,^^d^	
	*δ*_C_	*δ*_H_ (*J* in Hz)	*δ*_C_	*δ*_H_ (*J* in Hz)	*δ*_C_	*δ*_H_ (*J* in Hz)
1	30.3	2.74	29.9	2.82, t (7.4)	30.8	2.86, t (7.0)
2	46.4	2.74	46.0	2.73	41.7	2.81, t (7.0)
3	211.7		208.9		200.1	
4	48.7	2.67, dd (15.8, 7.1)	47.6	2.45, dd (15.9, 5.3)	131.1	6.07, d (15.8)
		2.55, dd (15.8, 7.1)		2.71, dd (15.9, 7.1)		
5	76.3	3.72, dt (12.1, 6.1)	76.8	3.69	147.0	6.77, dd (15.8, 6.9)
6	37.5	1.71	36.2	1.76	34.3	2.47
7	32.1	2.54	31.2	2.55, ddd (15.6, 9.8, 6.6)	33.8	2.64, t (7.1)
				2.63, ddd (15.6, 9.8, 5.8)		
8	65.6	3.42, qd (7.1, 2.0)	57.2	3.31, s	—	—
9	15.8	1.10, t (7.1)	—	—	—	—
1′	134.0		132.3		137.3	
2′	113.1	6.75, d (1.5)	105.1	6.39, s	105.6	6.39, s
3′	148.9		147.1		153.3	
4′	145.6		133.1		136.3	
5′	116.2	6.68, d (8.0)	147.1		153.3	
6′	121.8	6.58, dd (8.0, 1.5)	105.1	6.39, s	105.6	6.39, s
1′′	134.8		133.9		133.6	
2′′	113.2	6.73, d (1.6)	111.0	6.67, br s	115.5	6.63, br s
3′′	148.9		146.5		143.9	
4′′	145.8		143.9		142.1	
5′′	116.2	6.70, d (8.0)	114.4	6.82, d (7.9)	115.5	6.75, d (6.9)
6′′	121.8	6.61, dd (8.0, 1.6)	121.0	6.65, br d (7.9)	120.7	6.55, br d (6.9)
3′-OCH_3_	56.4	3.80, s	56.4	3.86, s	56.3	3.83, s
4′-OCH_3_	—	—	—	—	61.1	3.82, s
5′-OCH_3_	—	—	56.4	3.86, s	56.3	3.83, s
3′′-OCH_3_	56.4	3.82, s	56.0	3.87, s	—	—

a Measured in CD_3_OD.

b Measured in CDCl_3_.

c 400 MHz for ^1^H, 100 MHz for ^13^C.

d 600 MHz for ^1^H, 150 MHz for ^13^C.

Compound 5 showed a molecular formula of C_23_H_30_O_7_ by HR-ESI-MS (*m*/*z* 419.2060 [M + H]^+^, calcd 419.2070). A comparison of the spectral data (Table 2) of 5 and 4 revealed that an ethoxy group in 4 was changed to a methoxyl group in 5, which was confirmed by HMBC correlations from H_3_-8 (*δ*_H_ 3.31, s) to C-5 (*δ*_C_ 76.8). Moreover, an additional methoxyl group was substituted at C-5′ due to the equivalent aromatic protons [*δ*_H_ 6.39 (2H, s, H-2′, 6′)] and a symmetrical substituted aromatic ring (*δ*_C_ 132.3, 147.1 × 2, 105.1 × 2, 133.1). Similarly to 4, according to the specific optical rotation of 5 as [*α*]^25^_D_ −8.3, the absolute configuration of C-5 is assigned to *R*. To sum up, 5 was identified as (5*R*)-5-methoxy-1-(4-hydroxy-3,5-dimethoxy-phenyl)-7-(4-hydroxy-3-methoxyphenyl) heptan-3-one.

Compound 6 possessed a molecular formula of C_22_H_26_O_6_ based on HR-ESI-MS (*m*/*z* 387.1794 [M + H]^+^, calcd 387.1808). The ^1^H and ^13^C NMR data (Table 2) of 6 was high similarity to those of 20. The only difference between them was that a hydroxyl group in 20 was changed to a methoxyl group at C-4′ in 6, which was further deduced by HMBC correlations between 3.82 (3H, s, 4′-OCH_3_) and C-4′ (*δ*_C_ 136.3). Therefore, the structure of 6 was defined as (*E*)-7-(3,4-dihydroxyphenyl)-1-(3,4,5-trimethoxyphenyl) hept-4-en-3-one.

Compound 7 had the molecular formula of C_22_H_28_O_6_ inferred from the HR-ESI-MS at *m*/*z* 411.1767 ([M + Na]^+^, calcd 411.1784), corresponding to an index of hydrogen deficiency of 9. Its NMR data (Table 3) possessed similar signals to those of 10 except for the loss of one acetyl group located at C-5. The acetyl group was substituted at C-3, which was evidenced by HMBC correlations from H-9 (*δ*_H_ 2.03) to C-8 (*δ*_C_ 171.4), and H-3 (*δ*_H_ 4.90) to C-8 (*δ*_C_ 171.4). According to Brewster rule of secondary carbinol,^31–33^7 showed a negative optical rotation value of 10.5, which indicated that the acetyl moiety at C-3 is predicted to be *R* configuration. Therefore, 7 was determined and named as (3*R*)-3-acetoxy-7-(3,4-dihydroxy-phenyl)-1-(4-hydroxy-3-methoxyphenyl) heptane.

**Table 3:** ^1^H (600 MHz) and ^13^C (150 MHz) NMR data of compounds 7–8 in CDCl_3_

Pos.	7		8	
	*δ*_C_	*δ*_H_ (*J* in Hz)	*δ*_C_	*δ*_H_ (*J* in Hz)
1	31.6	2.58	32.1	2.59
		2.51, td (7.0, 2.7)		2.51, td (7.2, 2.0)
2	36.2	1.84	36.0	1.86
		1.76		1.76
3	73.8	4.90	73.5	4.92
4	34.0	1.56	33.8	1.56
5	24.7	1.30	24.7	1.29
6	31.1	1.51	30.9	1.49
7	35.0	2.46, td (7.5, 1.7)	34.9	2.4, t (7.6)
1′	133.7		132.9	
2′	111.2	6.66, br s	105.3	6.40, s
3′	146.5		147.0	
4′	143.8		132.9	
5′	114.5	6.83, d (7.9)	147.0	
6′	120.9	6.65	105.3	6.40, s
1′′	135.4		135.4	
2′′	115.5	6.64, br s	115.4	6.59, d (1.5)
3′′	143.5		143.4	
4′′	141.8		141.8	
5′′	115.2	6.76, d (7.6)	115.2	6.75, d (8.0)
6′′	121.0	6.56, br d (7.6)	120.9	6.56, dd (8.0, 1.5)
3-OAc	171.4		171.3	
	21.4	2.03, s	21.4	2.04, s
3′-OCH_3_	56.1	3.87, s	56.5	3.87, s
5′-OCH_3_	—	—	56.5	3.87, s

The molecular formula of compound 8 was determined as C_23_H_30_O_7_ by HR-ESI-MS (*m*/*z* 441.1895 [M + Na]^+^, calcd 441.1889). Extensive comparison of the NMR data of 8 and 7 (Table 3) suggested that an additional methoxyl group was located at C-5′ due to the equivalent aromatic protons [*δ*_H_ 6.40 (2H, s, H-2′, 6′)] and a symmetrical substituted aromatic ring (*δ*_C_ 147.0 × 2, 105.3 × 2, 132.9 × 2). Using the same method as above, it was determined that the absolute configuration of C-3 is *R* based on an optical rotation of [α]^25^_D_ −16.9. Thus, the structure of 8 was assigned, and named (3*R*)-3-acetoxy-7-(3,4-dihydroxyphenyl)-1-(4-hydroxy-3,5-dimethoxyphenyl)heptane.

The 15 known compounds (9–23) were identified as (3R*,5S*)-3,5-diacetoxy-1,7-bis(4-hydroxy-3-methoxyphenyl) heptane (9),^34^ (3S*,5S*)-3,5-diacetoxy-7-(3,4-dihydroxyphenyl)-1-(4-hydroxy-3-methoxyphenyl) heptane (10),^35^ (3R*,5S*)-3,5-diacetoxy-1-(4-hydroxy-3,5-dimethoxyphenyl)-7-(4-hydroxy-3-methoxyphenyl) heptane (11),^35^ (3S*,5S*)-3,5-diacetoxy-1-(4-hydroxy-3,5-dimethoxyphenyl)-7-(4-hydroxy-3-methoxyphenyl) heptane (12),^28^ hexahydrocurcumin (13),^36^ 5-acetyl hexahydro-curcumin D (14),^29^ 7-(3,4-dihydroxyphenyl)-1-(4-hydroxy-3-methoxyphenyl) heptan-3-one (15),^37^ platyphyllenone (16),^38^ isogingerenone C (17),^39^ gingerenone A (18),^29^ (*E*)-7-(3,4-dihydroxyphenyl)-1-(4-hydroxy-3-methoxyphenyl) hept-4-en-3-one (19),^40^ isogingerenone B (20),^41^ gingerenone B (21),^41^ (1S*,3R*,5S*) 3-acetoxy-1,5-epoxy-1-(3,4-dihydroxy-5-methoxy-phenyl)-7-(4-hydroxy-3-methoxyphenyl) heptane (22),^42^ and (1S*,3S*,5S*) 3-acetoxy-1,5-epoxy-1-(3,4-dihydroxy-5-methoxy-phenyl)-7-(4-hydroxy-3-methoxyphenyl) heptane (23).^42^

### 3.2. Predicted targets of isolated compounds

**Fig. 3:**
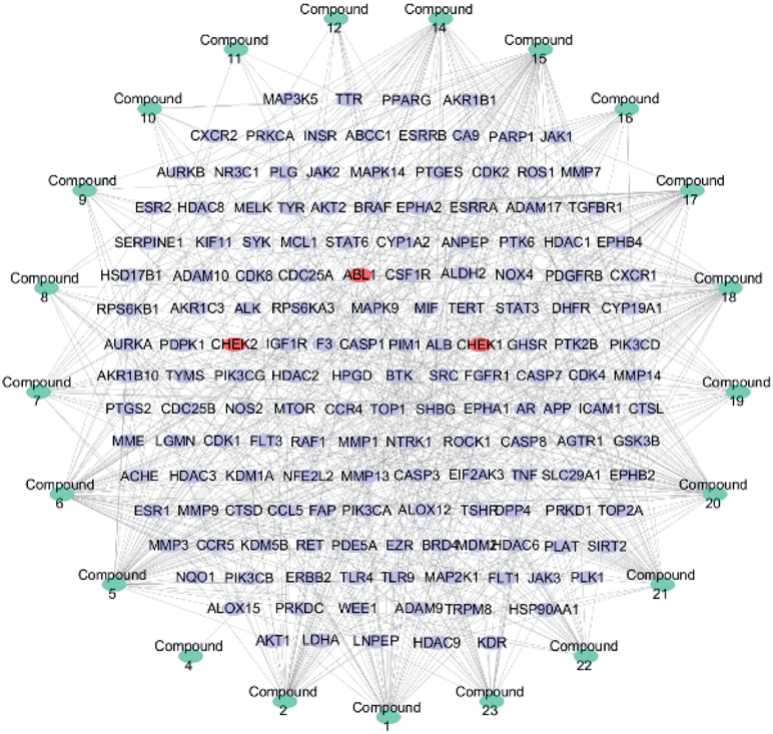
Potential anti-tumour target-compound network (blue stands for the compounds, purple represents the cancer related targets, and red means key targets).

**Fig. 4:**
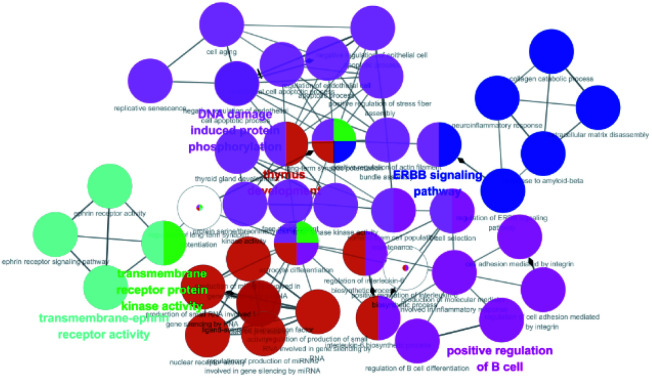
GO enrichment analysis of diarylheptanoids in the treatment of cancer.

357 targets were discovered as potential targets based on the probability in SwissTargetPrediction (3 and 13 did not have the potential targets). Then, 1381 cancer-related targets were extracted from GenCLiP3 with “cancer” as a keyword. Among them, there are 159 targets were duplicates. A compound-cancer target network consisting of 180 nodes (159 nodes of targets and 21 nodes of compounds) and 598 edges was constructed by using Cytoscape 3.5 software (Fig. 3). Thirty key targets were identified based on the intermediate value of degree, and their biological functions were associated with four pathways: DNA damage induced protein phosphorylation, ERGG pathway, positive regulation of B cell and transmembrane receptor protein kinases activity (Fig. 4). CHEK1 (CHK1), CHEK2 (CHK2) and ABL1 played an important role in DNA damage. Next, we focused on the protein phosphorylation induced by DNA damage pathway and conducted the activity verification.

### 3.3. Diarylheptanoids suppressed cell proliferation

All isolated diarylheptanoids were tested for their inhibitory activities against A549, HepG2, HeLa, MDA-MB-231, and HCT116 lines by using MTT method, and the results are shown in Table 4. Compounds 6, 16–19 showed cytotoxic effects against five human tumour cell lines with IC_50_ values ranging from 6.69–33.46 μM. Among them, compound 17 displayed more superior cytotoxicity against HCT116 cell lines with IC_50_ value of 6.69 ± 0.09 μM, compared to the positive control (curcumin, IC_50_: 14.88 ± 0.14 μM).

**Table 4:** Cytotoxicity activities of compounds 6, and 16–19

Compound	Cytotoxic activity IC_50_/μmol L^−1^				
	A549	HepG2	HeLa	MDA-MB-231	HCT116
6	17.49 ± 0.09	33.46 ± 0.50	30.46 ± 1.20	17.83 ± 0.11	14.70 ± 0.63
16	13.74 ± 0.48	19.67 ± 0.09	11.30 ± 0.36	10.78 ± 0.12	13.88 ± 0.18
17	11.49 ± 0.11	15.98 ± 0.14	10.41 ± 0.43	9.18 ± 0.18	10.61 ± 0.21
18	8.63 ± 0.42	13.06 ± 0.13	11.56 ± 0.21	10.51 ± 0.14	6.69 ± 0.09
19	17.27 ± 0.21	20.93 ± 0.41	12.20 ± 0.16	13.60 ± 0.05	11.64 ± 0.18
Curcumin^a^	21.86 ± 0.25	27.37 ± 0.30	19.71 ± 0.22	19.03 ± 0.11	14.88 ± 0.14

a Paclitaxel was used as a control.

The cytotoxic activity of compound 13 was nearly identical to compounds 4, 14 and 15, which suggested the cytotoxic activity of diarylheptanoids might be less dependent on hydroxy groups of heptane chain. The results of MTT assay for compounds 6, 16–19 indicated that the substituted phenyl ring might not be a critical structural determinant for the cytotoxic activities.^43^ The superior cytotoxic effect of 18 compared with 13 might be attributed to the presence of an α, β-unsaturated carbonyl moiety in 18, as reported earlier.^44,45^ Previously, it had been suggested that the α, β-unsaturated carbonyl compounds-mediated toxicity was attributable to formation of Michael-type adducts with the nucleophilic sulfhydryl groups of protein thiols.^46,47^ The above results were not sufficient to further clarify the structure–activity relationship between the diarylheptanoid derivatives and/or other components. More research may be required to clarify their potential selective cytotoxic activity.

### 3.4. Effects of diarylheptanoids on the ATR/CHK1 signalling pathway

Eukaryotic cells have such a cumbersome DNA damage response (DDR) to protect the integrity of the genome, which contains two main signal cascades, namely the ATM/CHK2 and ATR/CHK1 cascades. As the core protein and kinase of DDR, ATR can activate downstream CHK1 to form ATR/CHK1 signalling pathway, so that bodies have enough time to repair the wrong DNA before turning to the next stage or going to apoptosis. If the damage of the cell cannot be repaired, the DDR will lead the damaged cell to apoptosis.^48^

**Fig. 5:**
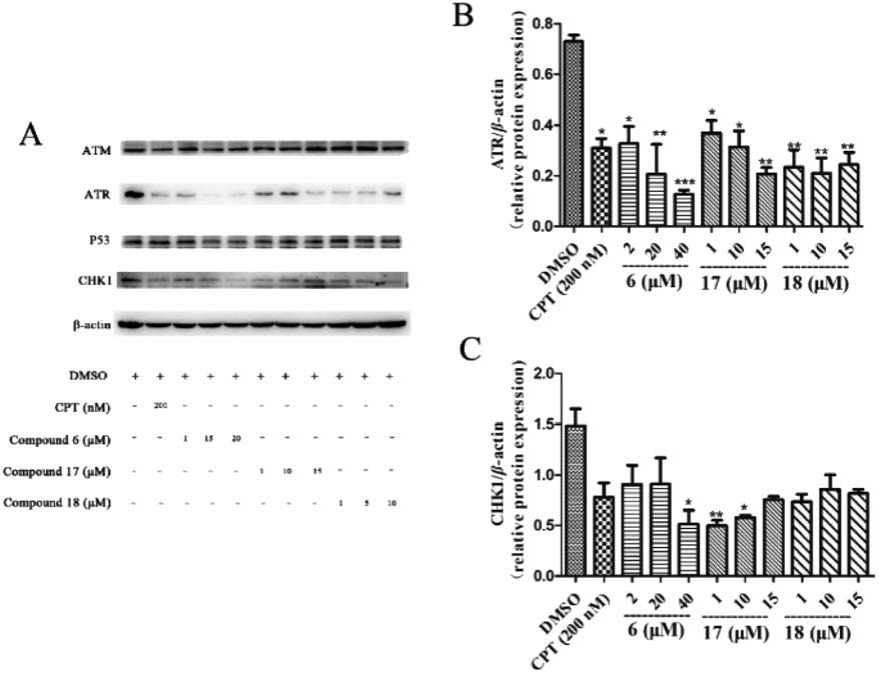
Effects of compounds 6, 17, and 18 on the protein expressions of ATM, ATR, P53, and CHK1 in HCT116 cell line. HCT116 cells were pre-treated with different concentrations of 6, 17, and 18 for 24 h. The cells were lysed with RIPA buffer and the protein levels for total ATM, ATR, P53, and CHK1 were measured by using immunoblot analysis. β-Actin was used as a loading control. CPT was used as a positive control. And all of the experiments have been repeated three times independently. Data presented as mean ± SD, *n* = 3. *p < 0.05, **p < 0.01, and ***p < 0.001 as compared with the DMSO group.

If the damage of the cell cannot be repaired, the DDR will lead the damaged cell to apoptosis.^48^ To understand the anti-tumour mechanism of diarylheptanoids in ginger, the effect of compounds 6, 17, and 18 (which showed superior suppressed cell proliferation activity) on the ATR/CHK1 signalling pathway was investigated. As shown in Fig. 5, compounds 6, 17, and 18 significantly suppressed the total protein levels of ATR and CHK1 in HCT116 cells. Our results indicated that diarylheptanoids had potential anti-tumour effects, and they might act through the regulation of the ATR/CHK1 signalling pathway.

## 4. Conclusions

In summary, 23 diarylheptanoids including eight undescribed ones (1–8), were isolated and identified from an important natural dietary ingredient ginger. It is worth mentioning that 1–3 are the first reported structures of diarylether heptanoids in *Z. officinale*, whereas 15–17 were isolated from *Zingiber* for the first time. Network pharmacology demonstrated that diarylheptanoids were mainly associated with the antitumor activity by affecting DNA damage signalling pathway. Cytotoxic and enzymatic activity assays discovered that compounds 6, 17, and 18 may exert anticancer effects by regulating the ATR/CHK1 signalling pathway.

## Conflicts of interest

The authors declare no conflict of interest.

## Supplementary Material

RA-011-D1RA03592D-s001

RA-011-D1RA03592D-s002
