# Supplementary material for: Diarylheptanoid analogues from the rhizomes of Zingiber officinale and their anti-tumour activity
Source: RSC Adv. 2021 Sep 2;11(47):29376–84. doi: 10.1039/d1ra03592d (PMC9040573; doi:10.1039/d1ra03592d)
Supplement: RA-011-D1RA03592D-s001 [file RA-011-D1RA03592D-s001.pdf]

Electronic Supplementary Material (ESI) for RSC Advances.
This journal is © The Royal Society of Chemistry 2021

Electronic Supplementary Information

Diaryheptanoid analogues from the rhizomes of *Zingiber officinale* and their anti-tumor activity

Ting Li,[a‡] Da-bo Pan,[a,b‡] Qian-qian Pang[a], Mi Zhou[a], Xiao-jun Yao[d], Xin-sheng Yao [a], Hai-bo Li [c, *], Yang Yu[a,*]

[a.] Institute of Traditional Chinese Medicine & Natural Products, and Guangdong Province Key Laboratory of Pharmacodynamic Constituents of TCM and New Drugs Research, College of Pharmacy, Jinan University, Guangzhou 510632, P. R. China Yang Yu, E-mail: 1018yuyang@163.com, Xinsheng Yao, E-mail: tyaoxs@jnu.eu.cn Tel: +86 20 85221767, Fax: +86 20 85221559

[b.] Department of Medical Technology, Qiandongnan Vocational & Technical College for Nationalities, Kaili, Guizhou 556000, P.R. china

[c.] Kanion Pharamaceutical Co. Ltd, State Key Laboratory of New-tech for Chinese Medicine Pharamaceutical Process, Lianyungang 222001, People' Repulic China.

[d.] State Key Laboratory of Applied Organic Chemistry, Department of Chemistry, Lanzhou University, Lanzhou 730000, P. R. China

[†] These authors contributed equally to this work.

1. Extraction and isolation of compounds 9-23

The ethyl acetate extract was subjected to silica gel column chromatography with cyclohexane/EtOAc (97:3, 95:5, 9:1, 8:2, 7:3, 6:4 and 0:1, v/v) to produce 22 subfractions (Fr. A-V). Fr. O was subjected to a ODS column (CH_3OH/H_2O 60%, v/v) to yield 11 subfractions (Fr. O1-O11). Fr. O8, O9, and O10 were purified by semi-preparative HPLC to yield compounds **16** (8.7 mg, 33% ACN/H_2O, v/v), **17** (16.1 mg, 33% ACN/H_2O, v/v), **18** (935.9 mg, 53% CH_3OH/H_2O, v/v), and **9** (13.2 mg, 38% ACN/H_2O, v/v), respectively. Fr. Q was chromatographed over a ODS column (CH_3OH/H_2O 60%, v/v) to yield 16 subfractions (Fr. Q1-Q16). Fr. Q6 and Q8 were further purified by semi-preparative HPLC to yield compounds **19** (48.0 mg, 25% ACN/H_2O, v/v) and **20** (5.0 mg, 33% ACN/H_2O, v/v), respectively. Fr. Q7 was preliminarily purified by semi-preparative HPLC (30% ACN/H_2O, v/v), and then further purified by semi-preparative HPLC to afford compounds **15** (14.3 mg, 32% ACN/H_2O, v/v), **22** (1.9 mg, 32% ACN/H_2O, v/v), **21** (1.1 mg, 32% ACN/H_2O, v/v), and **14** (1.1 mg, 35% ACN/H_2O, v/v). Similarly, Fr. R was put on a ODS column (CH_3OH/H_2O 60%, v/v) to yield 9 subfractions (Fr. R1-R9). Fr. R4 was further purified by semi-preparative HPLC to yield compound **13** (133.4 mg, 25% ACN/H_2O, v/v). Fr. R6 and R8 were subjected to a silica gel column (CH_2Cl_2/Aceton 95:5, v/v) individually, and further purified by semi-preparative HPLC to afford compounds **10** (5.0 mg, 33% ACN/H_2O, v/v), **23** (3.4 mg, 30% ACN/H_2O, v/v), **11** (25.6 mg, 36% ACN/H_2O, v/v), and **12** (31.1 mg, 33% ACN/H_2O, v/v), respectively.

2. Spectroscopic data of isolated new compounds

cyclogingerenone A (1) yellow oil; $[\alpha]_D^{25}$ +22.7 (*c* 0.3, CH_3OH); UV (MeOH) λ_{max} nm (log ε): 205 (4.32), 281 (3.56); IR (KBr) ν_{max} 3431, 2923, 2848, 1684, 1652, 1612, 1517, 1509, 1454, 1274, 1202, 1147, 1089, 1035 cm^{-1}; ^1H-NMR (600 MHz, in CD_3OD) and ^{13}C-NMR (150 MHz, in CD_3OD) data see Table 1; HR-ESI-MS (positive) *m/z* 355.1553 $[M+H]^+$ (calculated for $C_{21}H_{23}O_5$, 355.1545).

cyclogingerenone B (2) yellow oil; $[\alpha]_D^{25}$ -10.7 (*c* 0.2, CH_3OH); UV (MeOH) λ_{max} nm (log ε): 205 (4.15), 280 (3.23); IR (KBr) ν_{max} 3446, 2917, 2848, 1736, 1675, 1606, 1509,

1456, 1425, 1306,1268, 1237, 1208, 1156, 1083, 827 cm^{-1}; ^{1}H-NMR (600 MHz, in $CDCl_3$) and ^{13}C-NMR (150 MHz, in $CDCl_3$) data see Table 1; HR-ESI-MS (positive) *m/z* 355.1553 $[M+H]^+$ (calculated for $C_{21}H_{23}O_5$, 355.1545).

cyclogingerenone C (3) yellow oil; $[\alpha]_D^{25}$ +2.7 (*c* 2.5, $CHCl_3$); UV (MeOH) λ_{max} nm (log ε): 207 (4.30), 277 (3.42); IR (KBr) ν_{max} 3333, 2932, 2920, 2848, 1687, 1600, 1517, 1506, 1454, 1419, 1358, 1266, 1020, 1150, 1078, 1035, 839 cm^{-1}; ^{1}H-NMR (600 MHz, in $CDCl_3$) and ^{13}C-NMR (150 MHz, in $CDCl_3$) data see Table 1; HR-ESI-MS (positive) *m/z* 373.1654 $[M+H]^+$ (calculated for $C_{21}H_{25}O_6$, 373.1651).

(5R)-5-ethoxyhexahydrocurcumin (4) yellow oil; $[\alpha]_D^{25}$ -10.7 (*c* 0.6, $CHCl_3$); UV (MeOH) λ_{max} nm (log ε): 203 (4.19), 225 (3.94), 282 (3.59); IR (KBr) ν_{max} 3453, 2935, 1705, 1607, 1514, 1456, 1367, 1270, 1151, 813, 793 cm^{-1}; ^{1}H-NMR (600 MHz, in CD_3OD) and ^{13}C-NMR (150 MHz, in CD_3OD) data see Table 2; HR-ESI-MS (positive) *m/z* 403.2110 $[M+H]^+$ (calculated for $C_{23}H_{31}O_6$, 403.2121).

(5R)-5-methoxy-1-(4-hydroxy-3,5-dimethoxyphenyl)-7-(4-hydroxy-3-methoxyphenyl) heptan-3-one (5) yellow oil; $[\alpha]_D^{25}$ -8.3 (*c* 0.6, $CHCl_3$); UV (MeOH) λ_{max} nm (log ε): 203 (4.20), 278 (3.42), 351 (3.26); IR (KBr) ν_{max} 3443, 2981, 2848, 1739, 1515, 1460, 1428, 1271, 1240, 1116, 1033, 726 cm^{-1}; ^{1}H-NMR (600 MHz, in $CDCl_3$) and ^{13}C-NMR (1501 MHz, in $CDCl_3$) data see Table 2; HR-ESI-MS (positive) *m/z* 419.2060 $[M+H]^+$ (calculated for $C_{23}H_{31}O_7$, 419.2070).

(E)-7-(3,4-dihydroxyphenyl)-1-(3,4,5-trimethoxyphenyl) hept-4-en-3-one (6) yellow oil; UV (MeOH) λ_{max} nm (log ε): 205 (4.27), 278 (3.35); IR (KBr) ν_{max} 3479, 2932, 1713, 1698, 1591, 1511, 1456, 1425, 1280, 1240, 1127, 1006 cm^{-1}; ^{1}H-NMR (600 MHz, in $CDCl_3$) and ^{13}C-NMR (150 MHz, in $CDCl_3$) data see Table 2; HR-ESI-MS (positive) *m/z* 387.1794 $[M+H]^+$ (calculated for $C_{22}H_{27}O_6$, 387.1808).

(3R)-3-acetoxy-7-(3,4-dihydroxyphenyl)-1-(4-hydroxy-3-methoxyphenyl) heptane (7) yellow oil; $[\alpha]_D^{25}$ -10.5 (*c* 0.6, $CHCl_3$); UV (MeOH) λ_{max} nm (log ε): 202 (4.01), 280 (3.41); IR (KBr) ν_{max} 3444, 2932, 2858, 1728, 1719, 1515, 1459, 1376, 1267, 1024, 950 cm^{-1}; ^{1}H-NMR (600 MHz, in $CDCl_3$) and ^{13}C-NMR (150 MHz, in $CDCl_3$) data see

Table 3; HR-ESI-MS (positive) *m/z* 411.1767 $[M + Na]^+$ (calculated for $C_{22}H_{28}O_6Na$, 411.1784).

(3R)-3-acetoxy-7-(3,4-dihydroxyphenyl)-1-(4-hydroxy-3,5-dimethoxyphenyl) heptane (8) yellow oil; $[\alpha]_D^{25}$ -16.9 (*c* 0.6, $CHCl_3$); UV (MeOH) λ_{max} nm (log ε): 205 (4.37), 280 (3.53); IR (KBr) ν_{max} 3316, 2921, 2848, 1730, 1716, 1609, 1520, 1460, 1373, 1249, 1220, 1116 cm^{-1}; ^1H-NMR (600 MHz, in $CDCl_3$) and ^{13}C-NMR (150 MHz, in $CDCl_3$) data see Table 3; HR-ESI-MS (positive) *m/z* 387.1794 $[M + Na]^+$ (calculated for $C_{23}H_{30}O_7Na$, 387.1808).

Figure caption:

Figure S1. UV spectrum of compound **1**
Figure S2. HR-ESI-MS spectrum of compound **1**
Figure S3. IR spectrum of compound **1**
Figure S4. ^1H-NMR spectrum of compound **1**
Figure S5. ^{13}C-NMR spectrum of compound **1**
Figure S6. ^{13}C-NMR and DEPT 135 spectra of compound **1**
Figure S7. ^1H-^1H COSY spectrum of compound **1**
Figure S8. HSQC spectrum of compound **1**
Figure S9. HMBC spectrum of compound **1**
Figure S10. UV spectrum of compound **2**
Figure S11. HR-ESI-MS spectrum of compound **2**
Figure S12. IR spectrum of compound **2**
Figure S13. ^1H-NMR spectrum of compound **2**
Figure S14. ^{13}C-NMR spectrum of compound **2**
Figure S15. ^{13}C-NMR and DEPT 135 spectra of compound **2**
Figure S16. ^1H-^1H COSY spectrum of compound **2**
Figure S17. HSQC spectrum of compound **2**
Figure S18. HMBC spectrum of compound **2**
Figure S19. UV spectrum of compound **3**
Figure S20. HR-ESI-MS spectrum of compound **3**
Figure S21. IR spectrum of compound **3**
Figure S22. ^1H-NMR spectrum of compound **3**
Figure S23. ^{13}C-NMR spectrum of compound **3**
Figure S24. ^{13}C-NMR and DEPT 135 spectra of compound **3**
Figure S25. ^1H-^1H COSY spectrum of compound **3**
Figure S26. HSQC spectrum of compound **3**
Figure S27. HMBC spectrum of compound **3**
Figure S28. UV spectrum of compound **4**
Figure S29. HR-ESI-MS spectrum of compound **4**

Figure S30. IR spectrum of compound **4**
Figure S31. ^{1}H-NMR spectrum of compound **4**
Figure S32. ^{13}C-NMR spectrum of compound **4**
Figure S33. ^{13}C-NMR and DEPT 135 spectra of compound **4**
Figure S34. ^{1}H-^{1}H COSY spectrum of compound **4**
Figure S35. HSQC spectrum of compound **4**
Figure S36. HMBC spectrum of compound **4**
Figure S37. UV spectrum of compound **5**
Figure S38. HR-ESI-MS spectrum of compound **5**
Figure S39. IR spectrum of compound **5**
Figure S40. ^{1}H-NMR spectrum of compound **5**
Figure S41. ^{13}C-NMR spectrum of compound **5**
Figure S42. ^{1}H-^{1}H COSY spectrum of compound **5**
Figure S43. HSQC spectrum of compound **5**
Figure S44. HMBC spectrum of compound **5**
Figure S45. UV spectrum of compound **6**
Figure S46. HR-ESI-MS spectrum of compound **6**
Figure S47. IR spectrum of compound **6**
Figure S48. ^{1}H-NMR spectrum of compound **6**
Figure S49. ^{13}C-NMR spectrum of compound **6**
Figure S50. ^{1}H-^{1}H COSY spectrum of compound **6**
Figure S51. HSQC spectrum of compound **6**
Figure S52. HMBC spectrum of compound **6**
Figure S53. UV spectrum of compound **7**
Figure S54. HR-ESI-MS spectrum of compound **7**
Figure S55. IR spectrum of compound **7**
Figure S56. ^{1}H-NMR spectrum of compound **7**
Figure S57. ^{13}C-NMR spectrum of compound **7**
Figure S58. ^{1}H-^{1}H COSY spectrum of compound **7**
Figure S59. HSQC spectrum of compound **7**
Figure S60. HMBC spectrum of compound **7**
Figure S61. UV spectrum of compound **8**
Figure S62. HR-ESI-MS spectrum of compound **8**
Figure S63. IR spectrum of compound **8**
Figure S64. ^{1}H-NMR spectrum of compound **8**
Figure S65. ^{13}C-NMR spectrum of compound **8**
Figure S66. ^{13}C-NMR and DEPT 135 spectra of compound **8**
Figure S67. ^{1}H-^{1}H COSY spectrum of compound **8**
Figure S68. HSQC spectrum of compound **8**
Figure S69. HMBC spectrum of compound **8**
Figure S70. The structures of compounds **3a** and **3b.**
Figure S71. Experimental and calculated CD spectra of compound **3Figure S72.** Effects of compounds **6**, **17**, and **18** on the protein expression of ATM, ATR, P53, and CHK1 in A549 cell line. A549 cells were pretreated with different concentrations of

compounds **6**, **17**, and **18** for 24 h. The cells were lysed with RIPA buffer and the protein levels for total ATM, ATR, P53, and CHK1 were measured by using immunoblot analysis. *β*-Actin was used as a loading control. CPT was used as a positive control. And all of the experiments were repeated three times independently.

3. Spectra of compounds 1-8

Compound 1

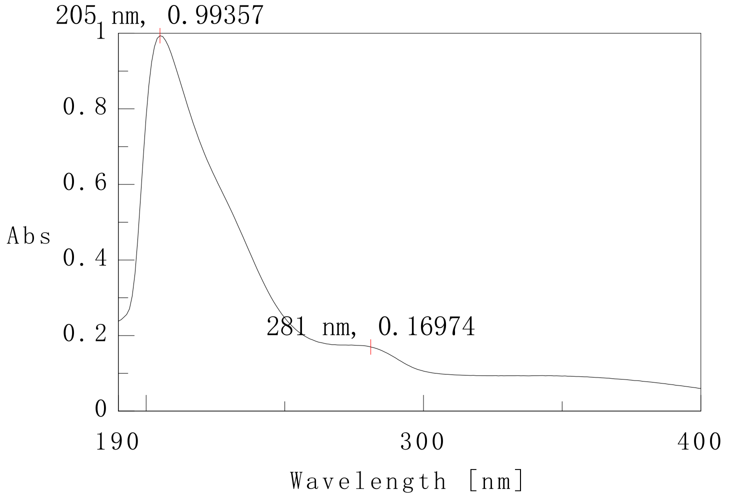

Figure S1. UV spectrum of compound 1

Elemental Composition Report — Page 1

Single Mass Analysis
Tolerance = 5.0 PPM / DBE: min = -1.5, max = 50.0
Element prediction: Off
Number of isotope peaks used for i-FIT = 3

Monoisotopic Mass, Even Electron Ions
335 formula(e) evaluated with 1 results within limits (all results (up to 1000) for each mass)
Elements Used:
C: 0-55 H: 0-100 N: 0-8 O: 0-8
Z0207-1B
2019031122 188 (1.518)

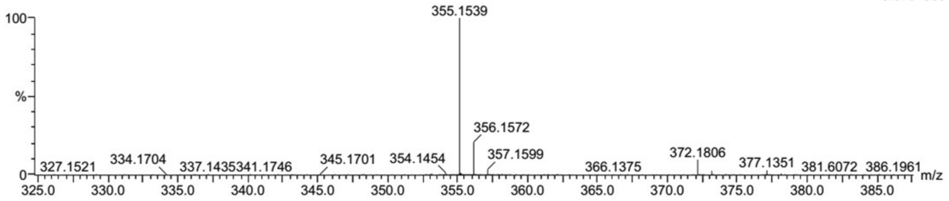

Minimum:				-1.5			
Maximum:		10.0	5.0	50.0			
Mass	Calc. Mass	mDa	PPM	DBE	i-FIT	Conf(%)	Formula
355.1539	355.1545	-0.6	-1.7	10.5	675.0	n/a	C21 H23 O5

Figure S2. HR-ESI-MS spectrum of compound 1

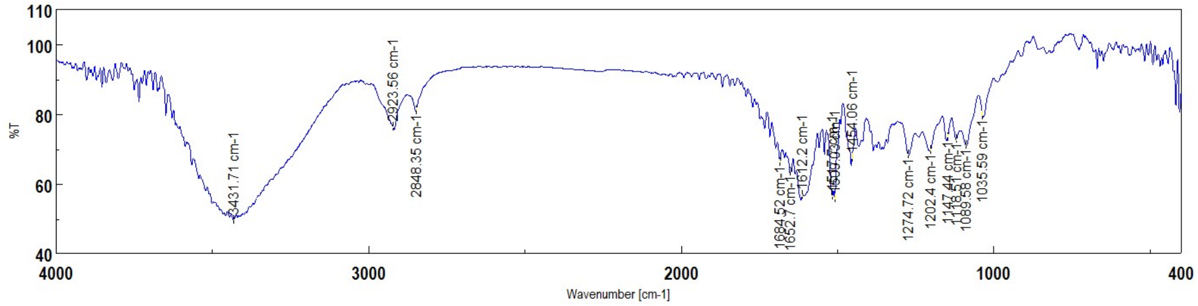

Figure S3. IR spectrum of compound 1

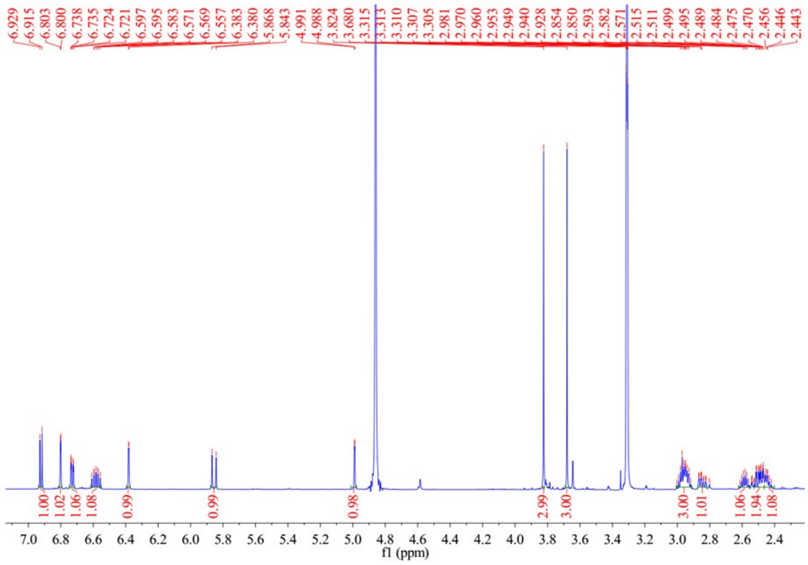

Figure S4. ^{1}H-NMR spectrum of compound 1

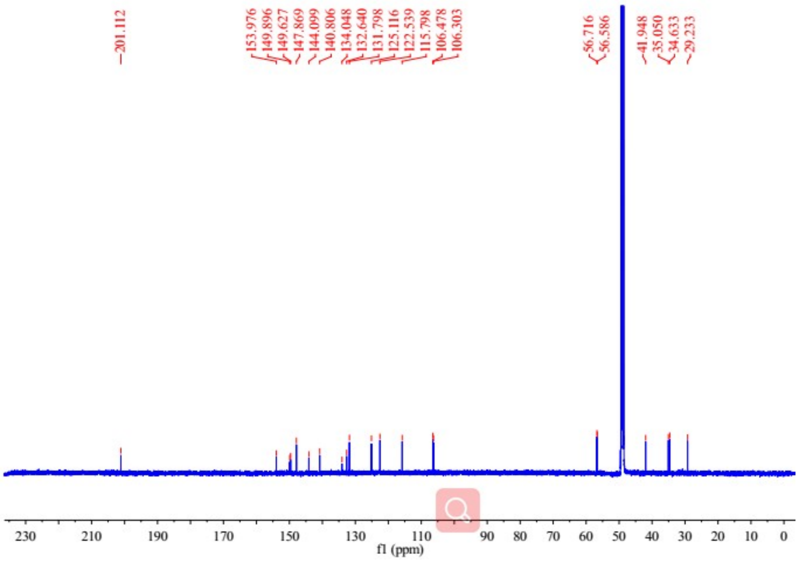

Figure S5. ^{13}C-NMR spectrum of compound 1

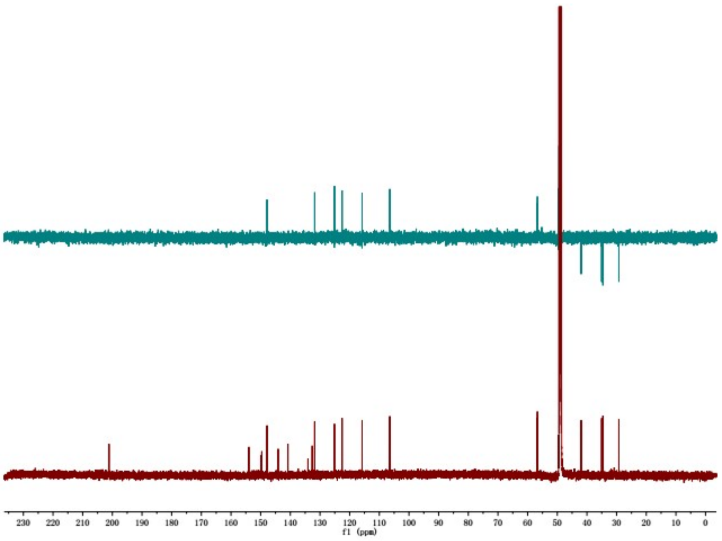

Figure S6. ^{13}C-NMR and DEPT 135 spectra of compound 1

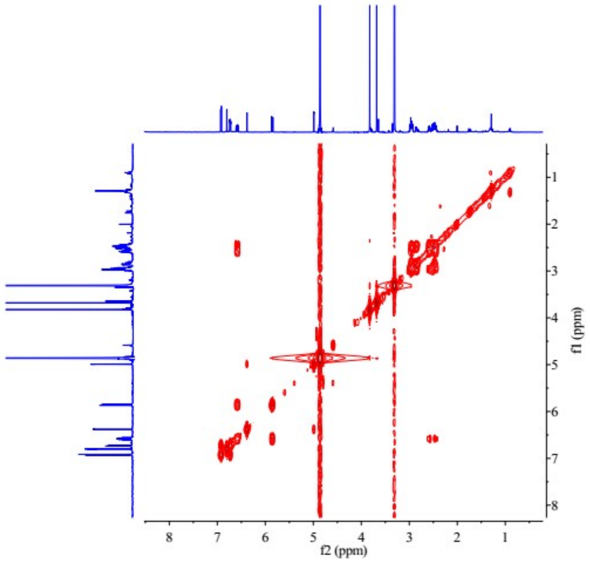

Figure S7. ^{1}H-^{1}H COSY spectrum of compound 1

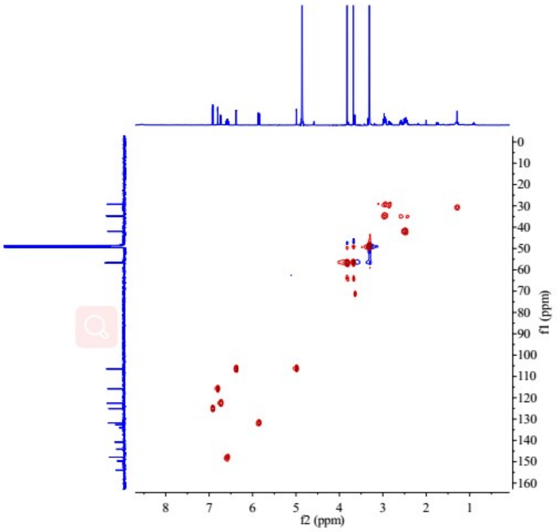

Figure S8. HSQC spectrum of compound 1

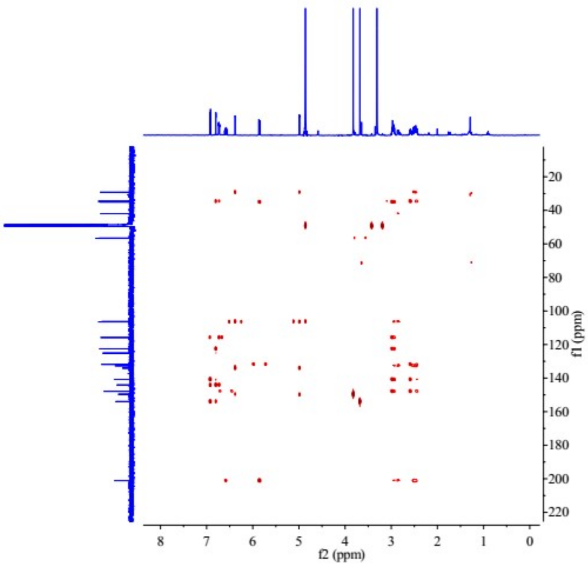

Figure S9. HMBC spectrum of compound 1

Compound 2

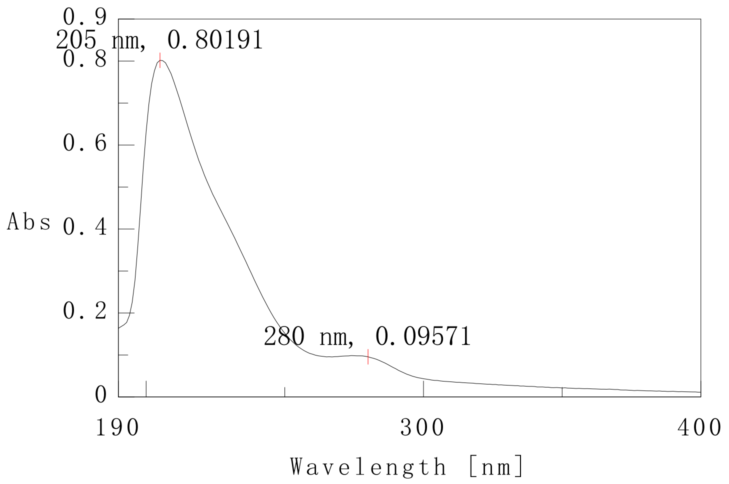

Figure S10. UV spectrum of compound 2

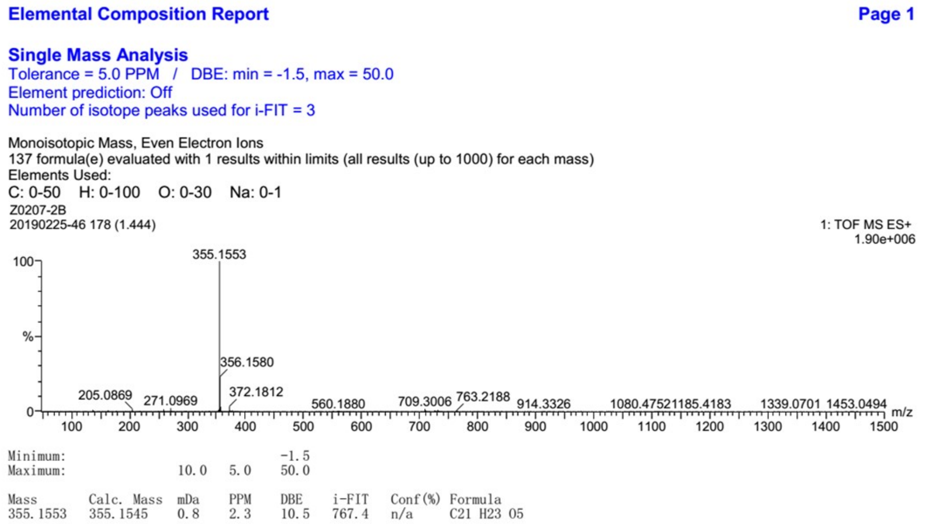

Figure S11. HR-ESI-MS spectrum of compound 2

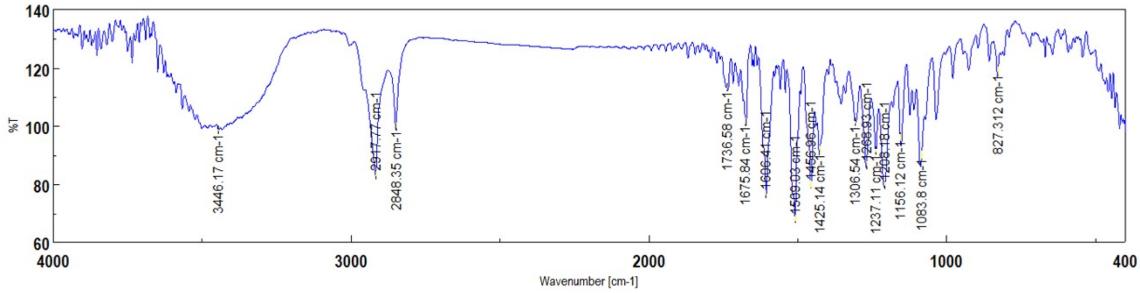

Figure S12. IR spectrum of compound 2

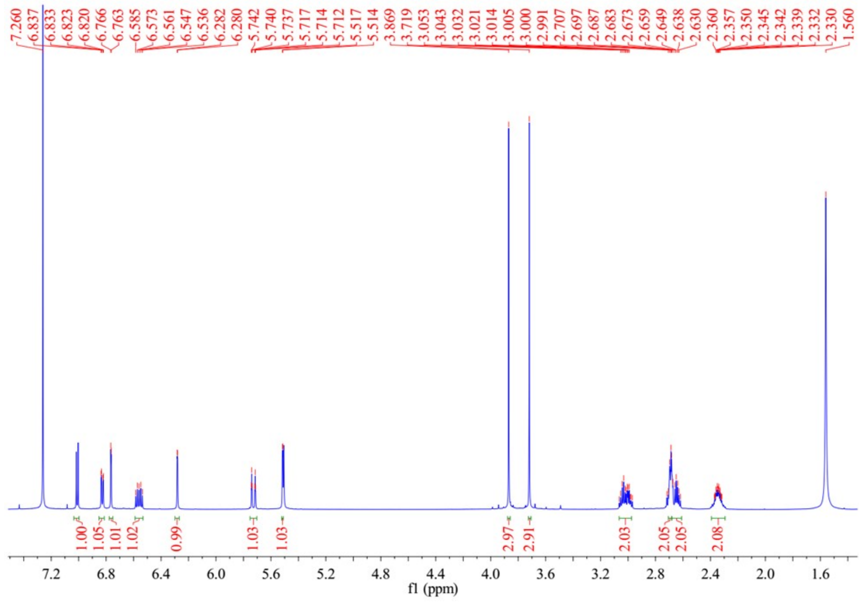

Figure S13. ^{1}H-NMR spectrum of compound 2

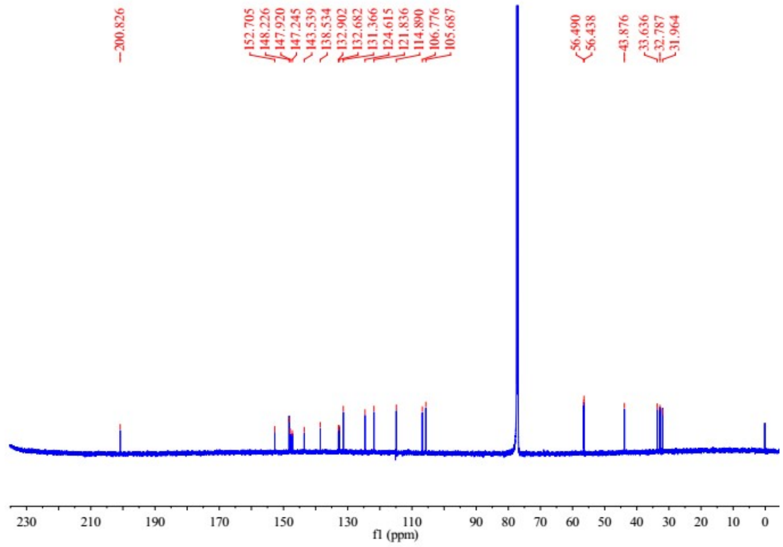

Figure S14. ^{13}C-NMR spectrum of compound 2

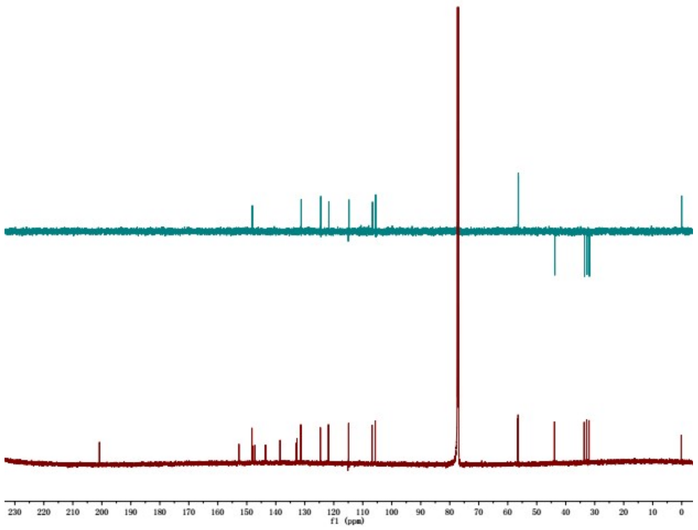

Figure S15. ^{13}C-NMR and DEPT 135 spectra of compound 2

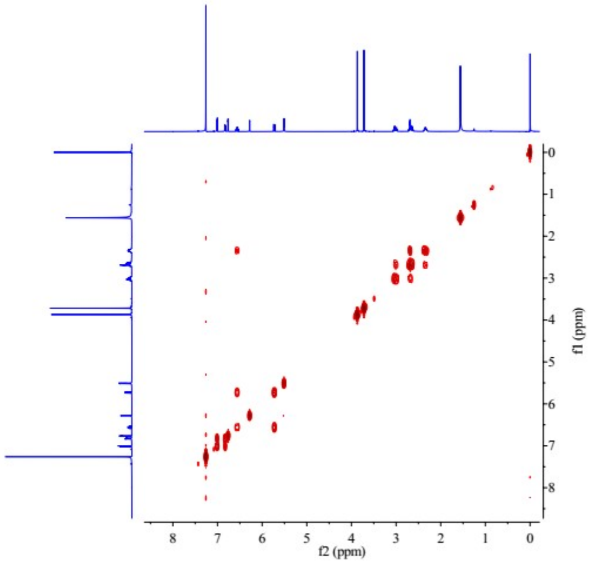

Figure S16. ^{1}H-^{1}H COSY spectrum of compound 2

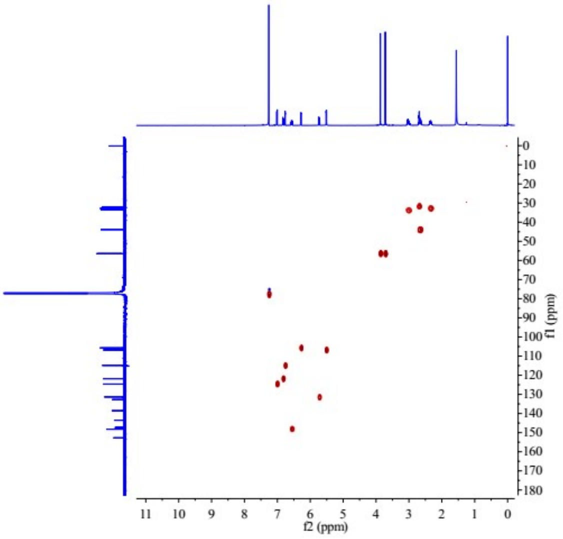

Figure S17. HSQC spectrum of compound 2

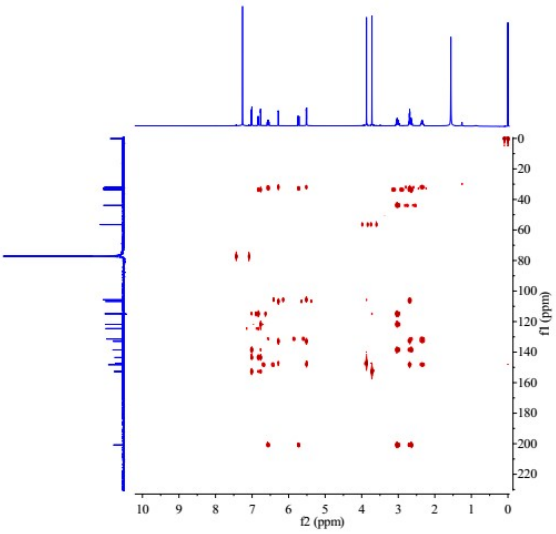

Figure S18. HMBC spectrum of compound 2

Compound 3

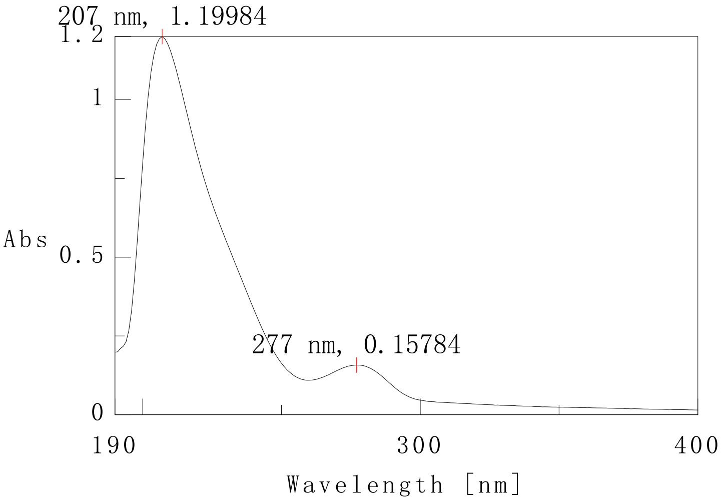

Figure S19. UV spectrum of compound 3

Elemental Composition Report **Page 1**

Single Mass Analysis
Tolerance = 5.0 mDa / DBE: min = -1.5, max = 50.0
Element prediction: Off
Number of isotope peaks used for i-FIT = 3

Monoisotopic Mass, Even Electron Ions
77 formula(e) evaluated with 1 results within limits (up to 50 best isotopic matches for each mass)
Elements Used:
C: 0-500 H: 0-1000 O: 0-200

Z02R4G2
20191118010 153 (1.246) 1: TOF MS ES+
6.52e+005

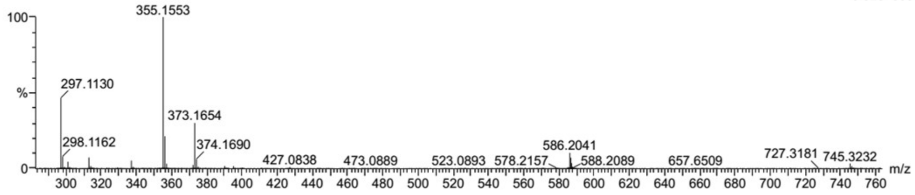

Minimum:				-1.5			
Maximum:		5.0	10.0	50.0			
Mass	Calc. Mass	mDa	PPM	DBE	i-FIT	Conf(%)	Formula
373.1654	373.1651	0.3	0.8	9.5	354.1	n/a	C21 H25 O6

Figure S20. HR-ESI-MS spectrum of compound 3

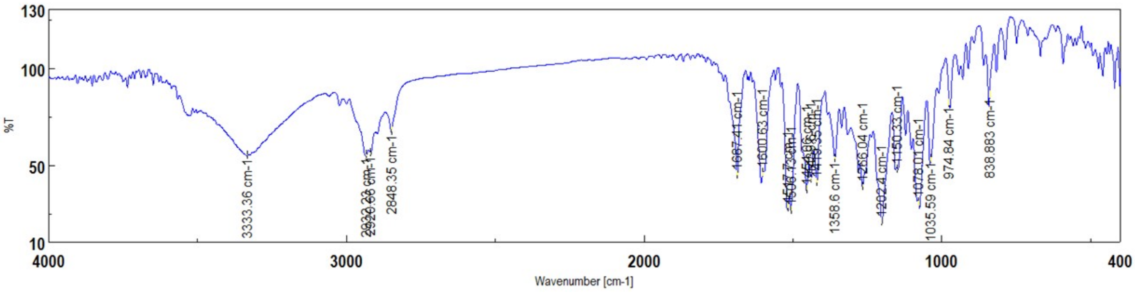

Figure S21. IR spectrum of compound 3

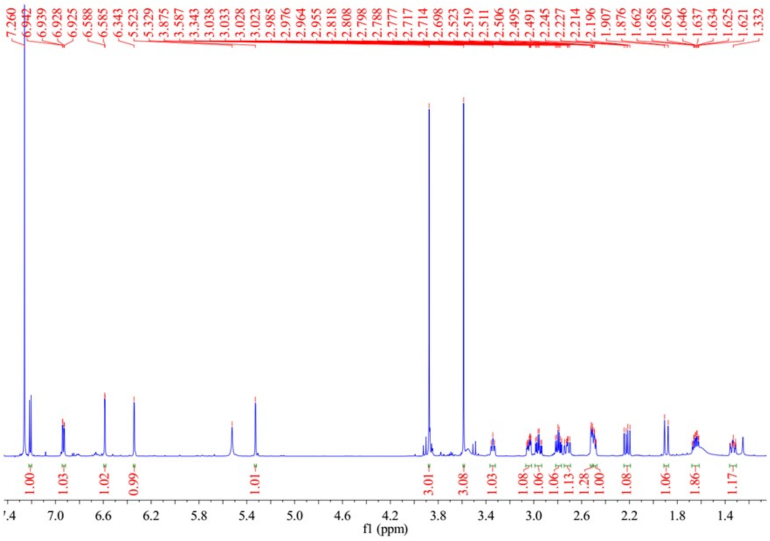

Figure S22. ^{1}H-NMR spectrum of compound 3

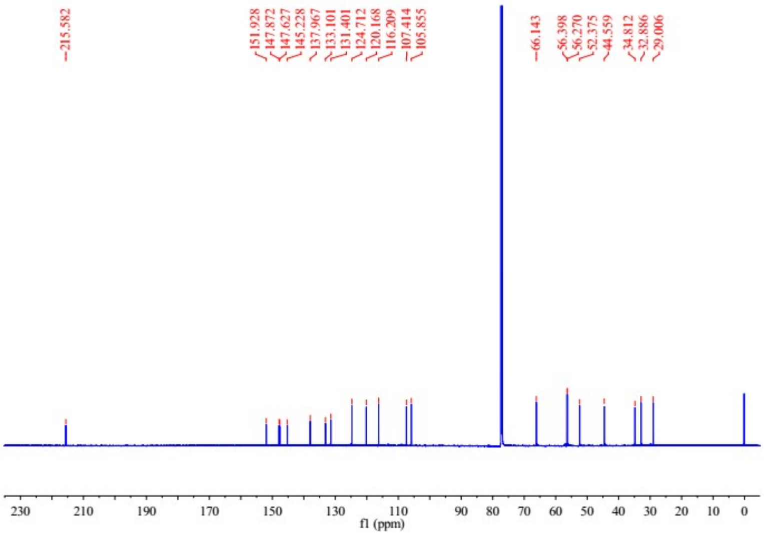

Figure S23. ^{13}C-NMR spectrum of compound 3

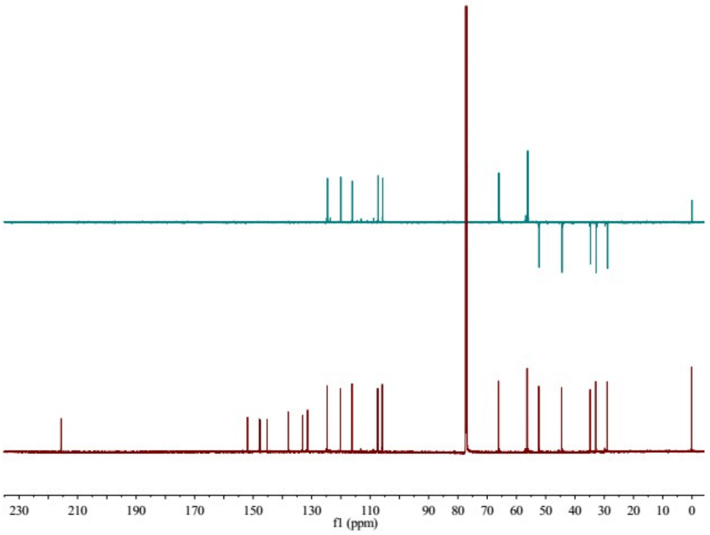

Figure S24. ^{13}C-NMR and DEPT 135 spectra of compound 3

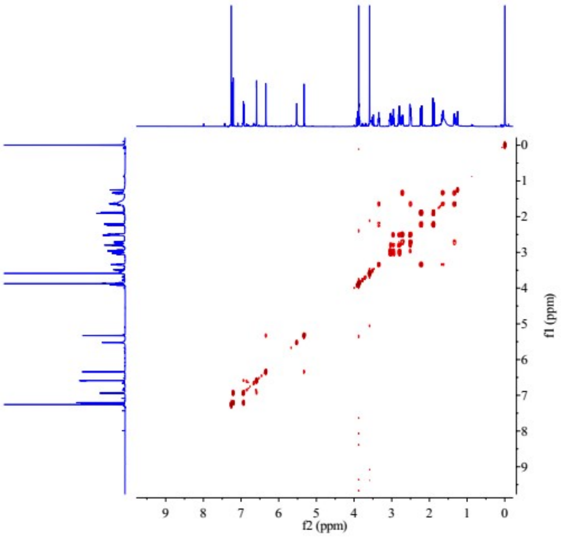

Figure S25. ^{1}H-^{1}H COSY spectrum of compound 3

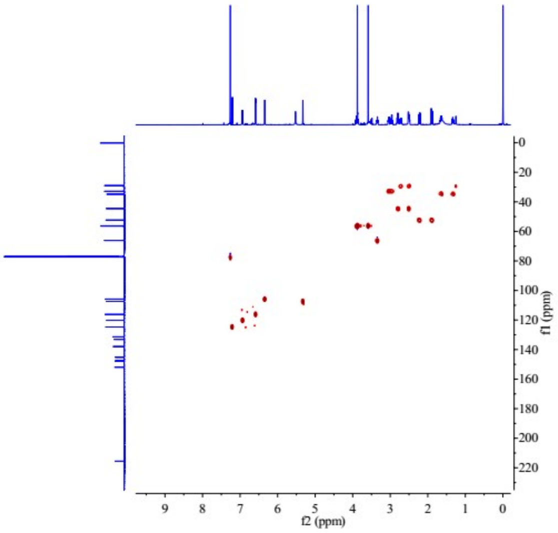

Figure S26. HSQC spectrum of compound 3

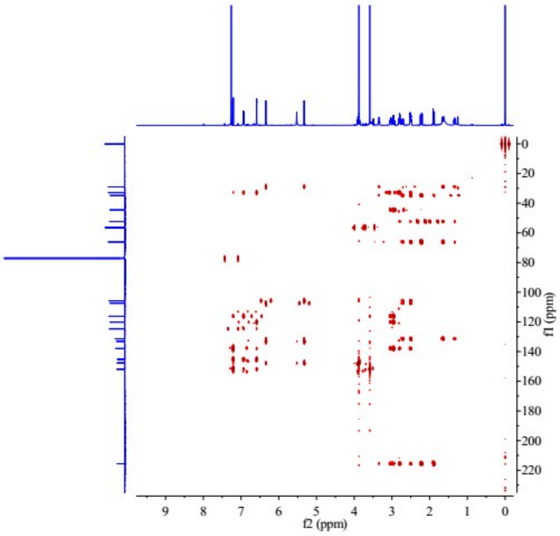

Figure S27. HMBC spectrum of compound 3

Compound 4

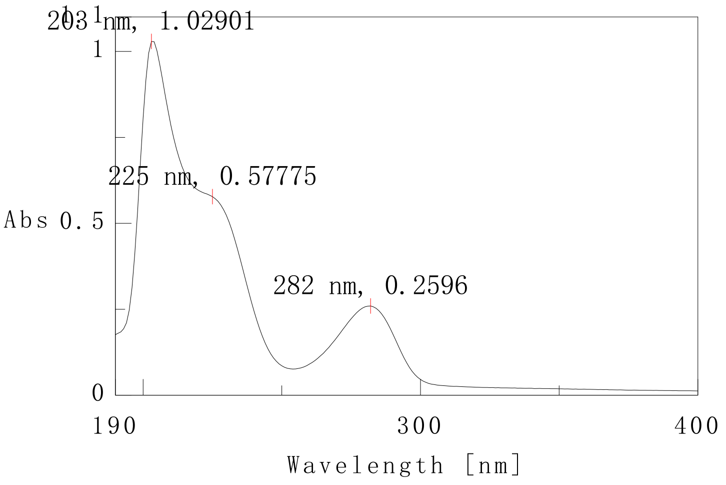

Figure S28. UV spectrum of compound 4

Elemental Composition Report Page 1

Single Mass Analysis
Tolerance = 10.0 PPM / DBE: min = -1.5, max = 50.0
Element prediction: Off
Number of isotope peaks used for i-FIT = 3

Monoisotopic Mass, Even Electron Ions
92 formula(e) evaluated with 1 results within limits (all results (up to 1000) for each mass)
Elements Used:
C: 0-60 H: 0-100 O: 0-30
ZO209C1
20190610008 195 (1.582)

1: TOF MS ES+
1.05e+005

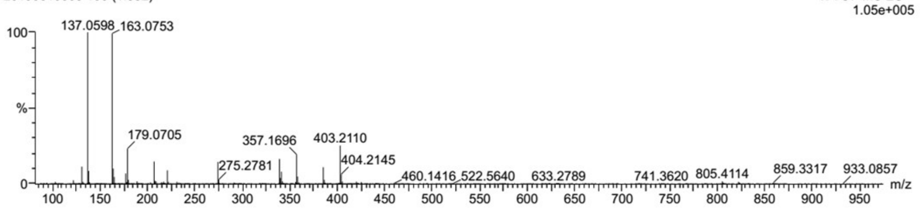

Minimum:				-1.5			
Maximum:		10.0	10.0	50.0			
Mass	Calc. Mass	mDa	PPM	DBE	i-FIT	Conf(%)	Formula
403.2110	403.2121	-1.1	-2.7	8.5	156.5	n/a	C23 H31 O6

Figure S29. HR-ESI-MS spectrum of compound 4

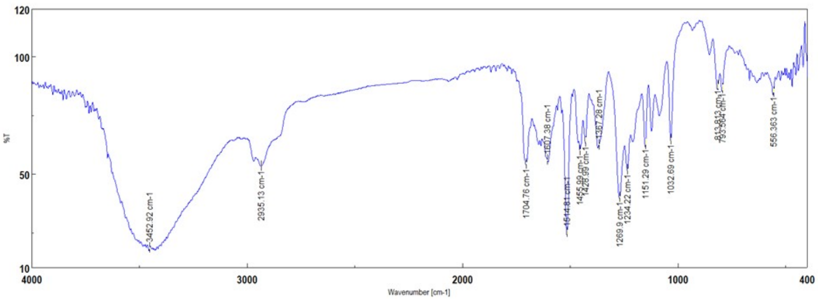

Figure S30. IR spectrum of compound 4

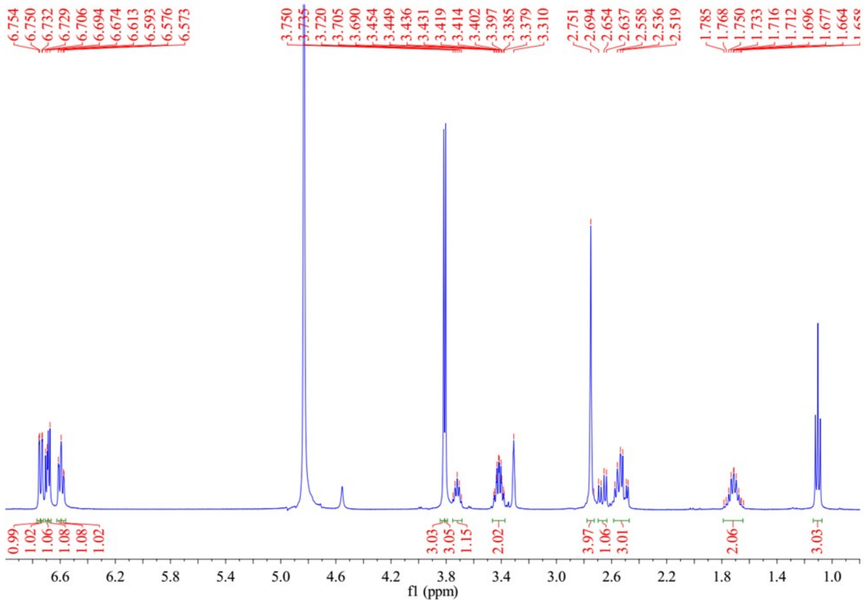

Figure S31. ^{1}H-NMR spectrum of compound 4

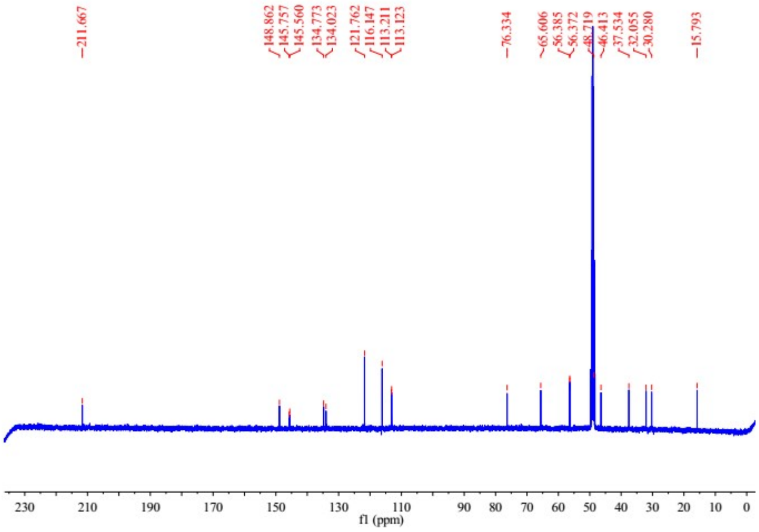

Figure S32. ^{13}C-NMR spectrum of compound 4

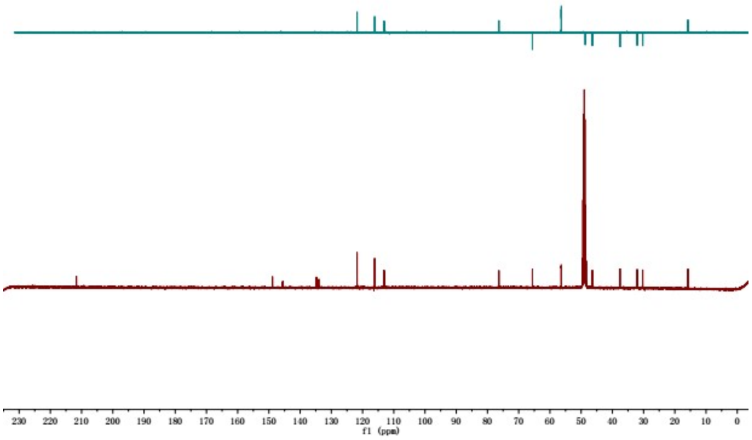

Figure S33. ^{13}C-NMR and DEPT 135 spectra of compound 4

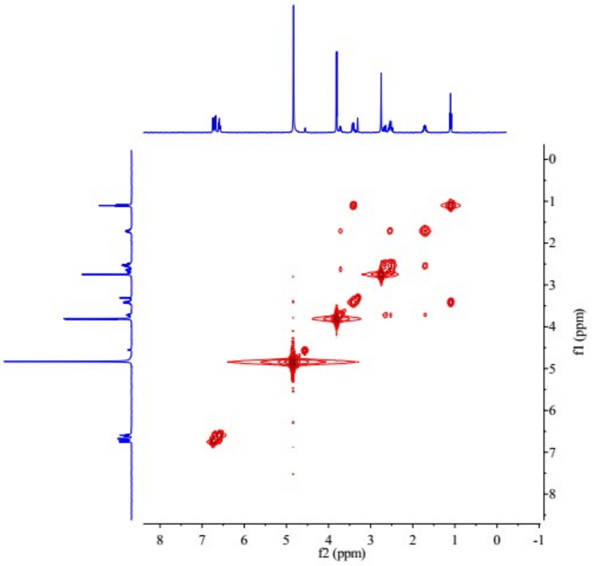

Figure S34. ^{1}H-^{1}H COSY spectrum of compound 4

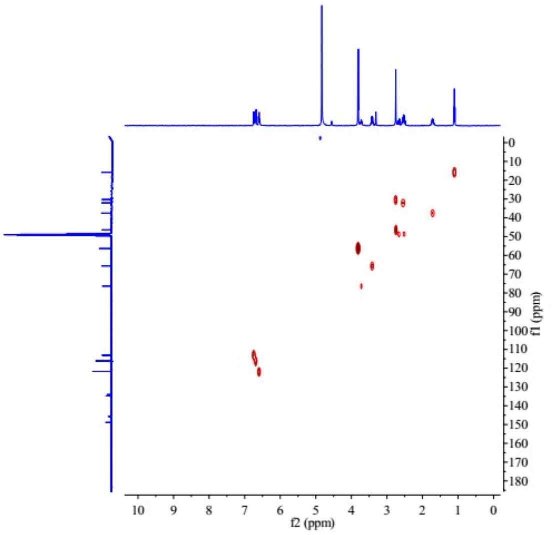

Figure S35. HSQC spectrum of compound 4

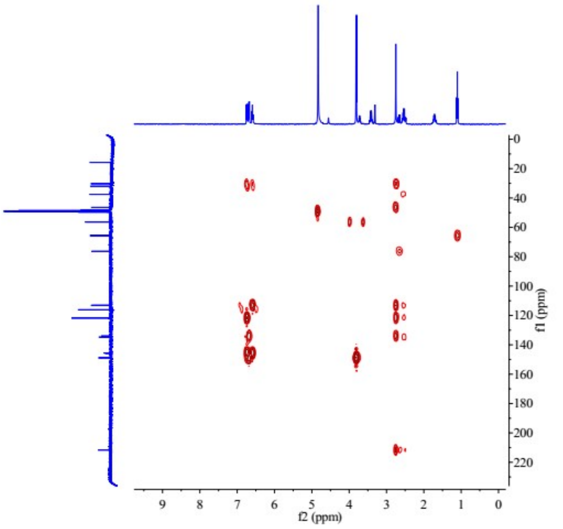

Figure S36. HMBC spectrum of compound 4

Compound 5

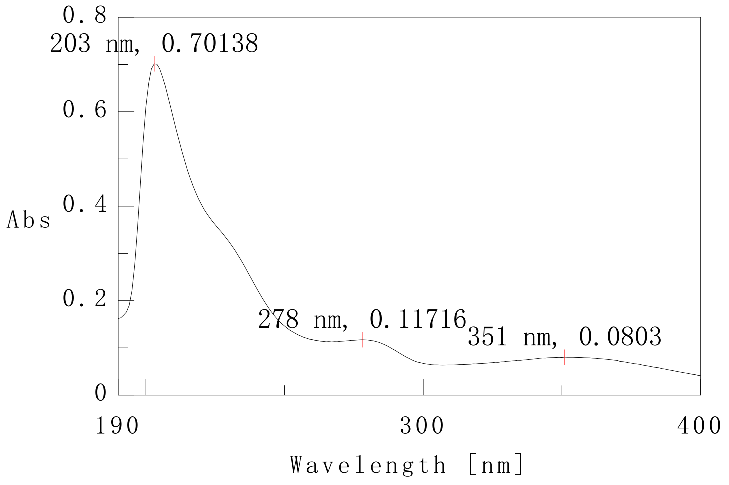

Figure S37. UV spectrum of compound 5

Elemental Composition Report Page 1

Single Mass Analysis
Tolerance = 10.0 PPM / DBE: min = -1.5, max = 50.0
Element prediction: Off
Number of isotope peaks used for i-FIT = 3

Monoisotopic Mass, Even Electron Ions
98 formula(e) evaluated with 1 results within limits (all results (up to 1000) for each mass)
Elements Used:
C: 0-50 H: 0-80 O: 0-30
Z02Q7I1
20191028-29 181 (1.466) 1: TOF MS ES+ 2.92e+004

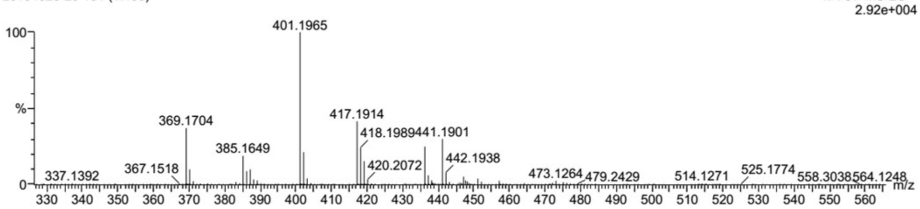

	Calc. Mass	mDa	PPM	DBE	i-FIT	Conf(%)	Formula
Minimum:				-1.5			
Maximum:		10.0	10.0	50.0			
Mass	Calc. Mass	mDa	PPM	DBE	i-FIT	Conf(%)	Formula
419.2060	419.2070	-1.0	-2.4	8.5	109.0	n/a	C23 H31 O7

Figure S38. HR-ESI-MS spectrum of compound 5

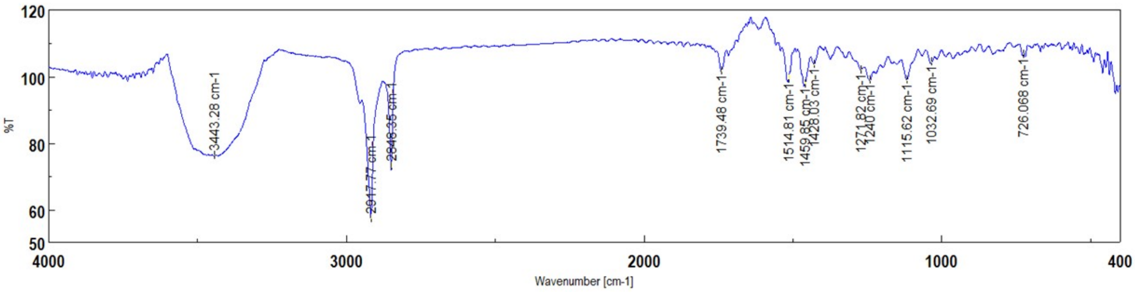

Figure S39. IR spectrum of compound 5

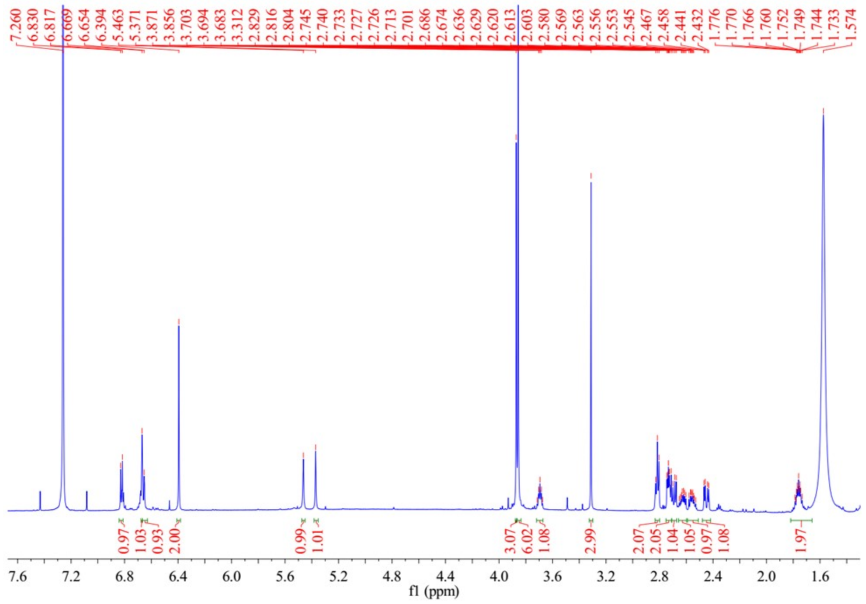

Figure S40. ^{1}H-NMR spectrum of compound 5

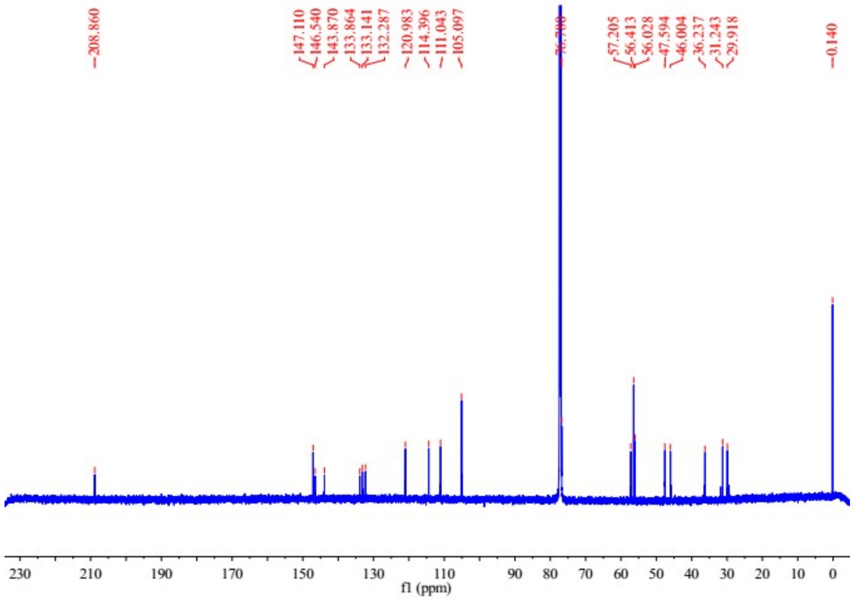

Figure S41. ^{13}C-NMR spectrum of compound 5

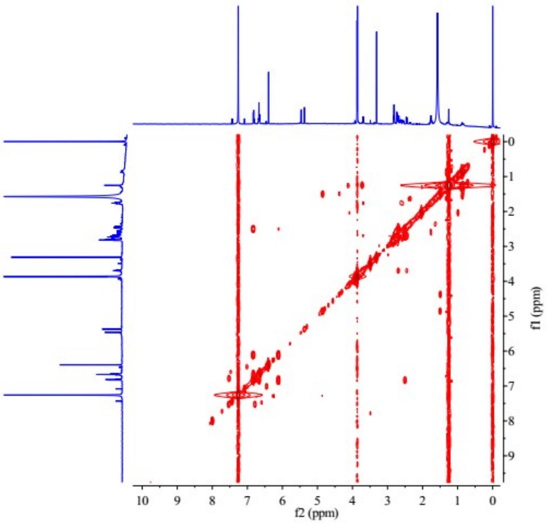

Figure S42. ^{1}H-^{1}H COSY spectrum of compound 5

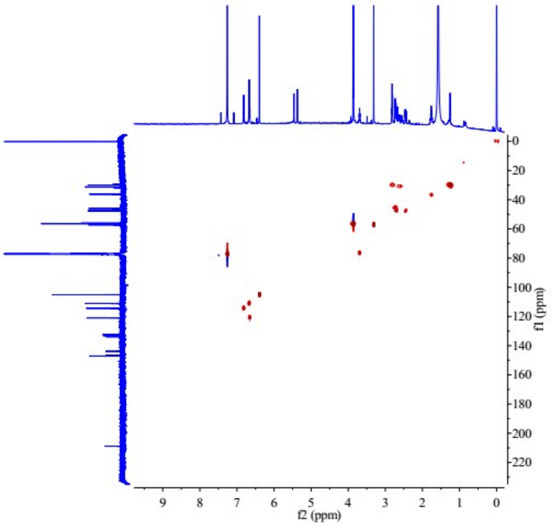

Figure S43. HSQC spectrum of compound 5

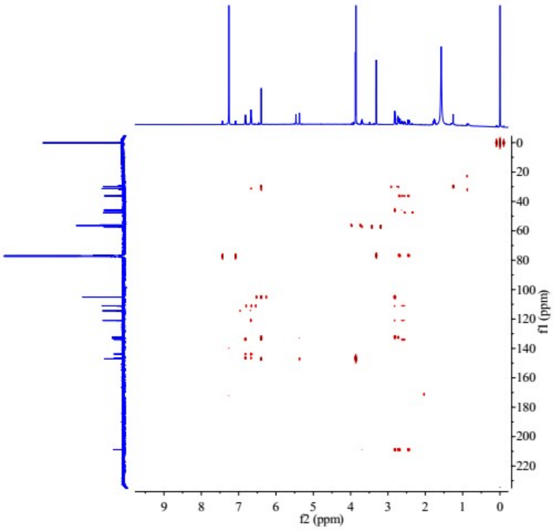

Figure S44. HMBC spectrum of compound 5

Compound 6

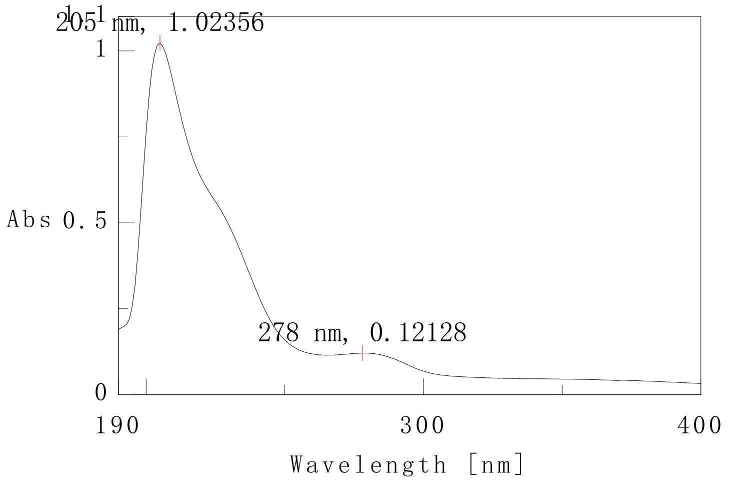

Figure S45. UV spectrum of compound 6

Elemental Composition Report **Page 1**

Single Mass Analysis
Tolerance = 5.0 mDa / DBE: min = -1.5, max = 50.0
Element prediction: Off
Number of isotope peaks used for i-FIT = 3

Monoisotopic Mass, Even Electron Ions
86 formula(e) evaluated with 2 results within limits (up to 50 best isotopic matches for each mass)
Elements Used:
C: 0-500 H: 0-1000 O: 0-200
Z02Q7M2
20191028-27 186 (1.503)

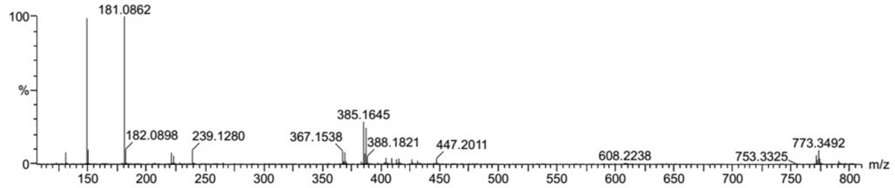

Minimum:				-1.5			
Maximum:		5.0	10.0	50.0			
Mass	Calc. Mass	mDa	PPM	DBE	i-FIT	Conf(%)	Formula
387.1794	387.1808	-1.4	-3.6	9.5	224.0	97.71	C22 H27 O6
	387.1749	4.5	11.6	18.5	227.7	2.29	C29 H23 O

Figure S46. HR-ESI-MS spectrum of compound 6

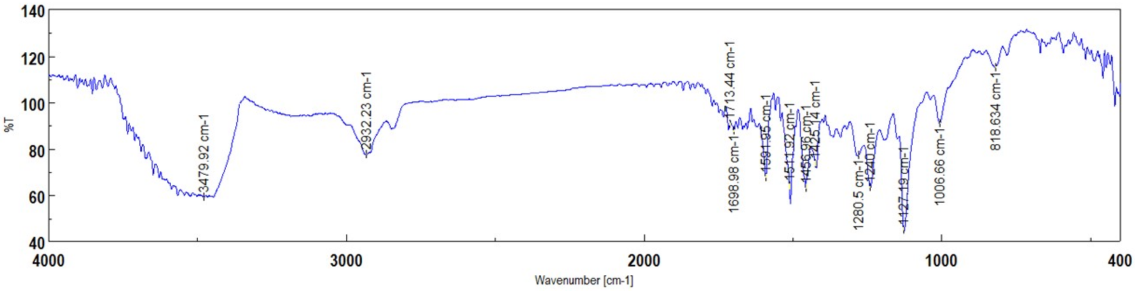

Figure S47. IR spectrum of compound 6

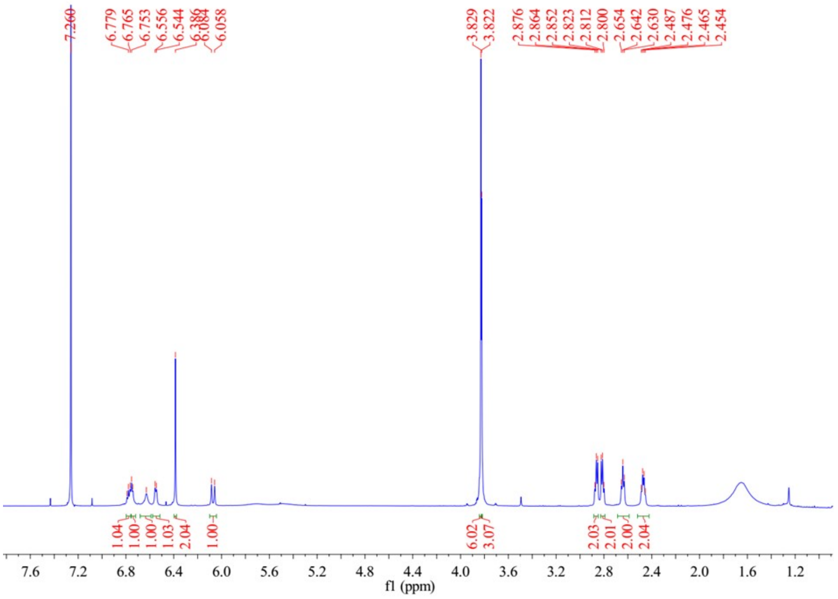

Figure S48. ^{1}H-NMR spectrum of compound 6

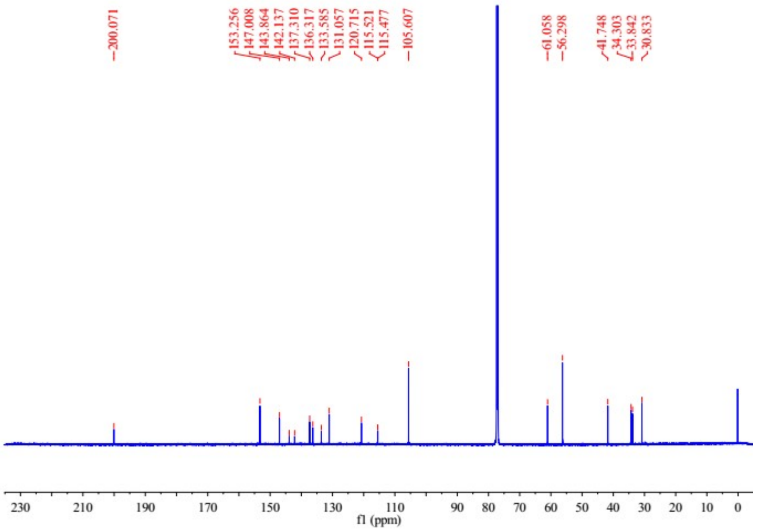

Figure S49. ^{13}C-NMR spectrum of compound 6

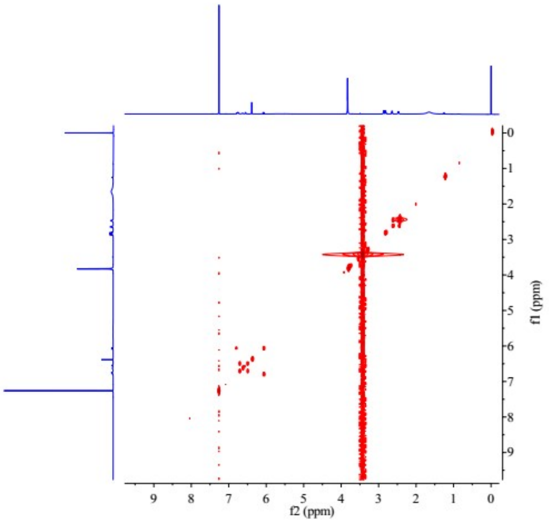

Figure S50. ^{1}H-^{1}H COSY spectrum of compound 6

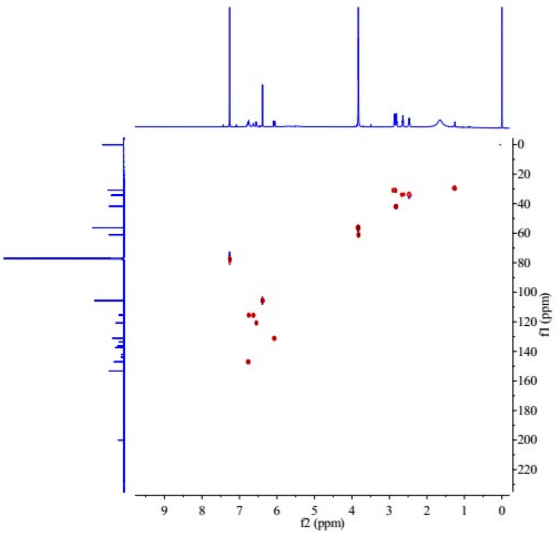

Figure S51. HSQC spectrum of compound 6

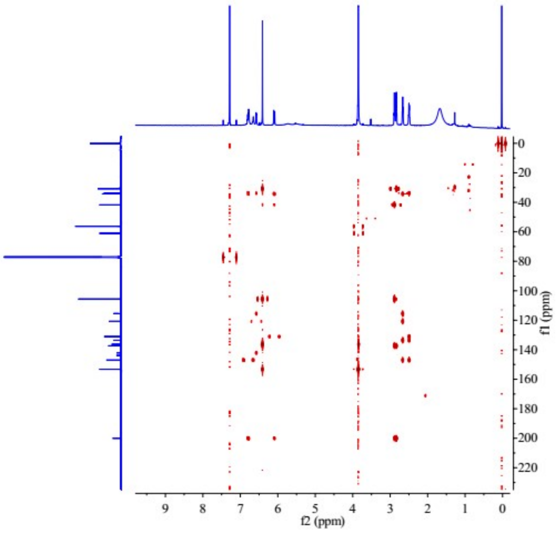

Figure S52. HMBC spectrum of compound 6

Compound 7

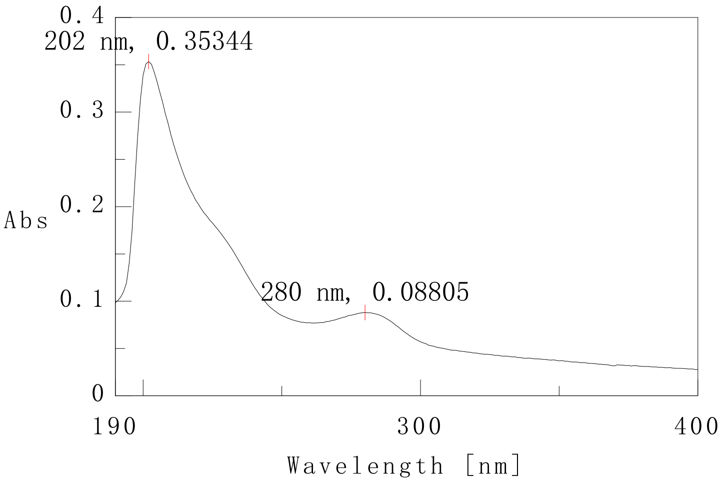

Figure S53. UV spectrum of compound7

Elemental Composition Report Page 1

Single Mass Analysis
Tolerance = 10.0 PPM / DBE: min = -1.5, max = 50.0
Element prediction: Off
Number of isotope peaks used for i-FIT = 3

Monoisotopic Mass, Even Electron Ions
177 formula(e) evaluated with 3 results within limits (all results (up to 1000) for each mass)
Elements Used:
C: 0-100 H: 0-500 O: 0-50 Na: 0-1
ZO2Q10F
20200610014 164 (1.328) 1: TOF MS ES+ 7.57e+004

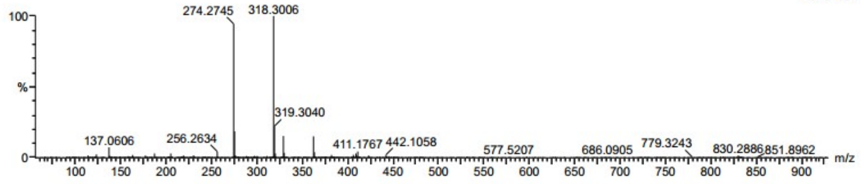

Minimum:				-1.5			
Maximum:		5.0	10.0	50.0			
Mass	Calc. Mass	mDa	PPM	DBE	i-FIT	Conf(%)	Formula
411.1767	411.1784	-1.7	-4.1	8.5	89.1	58.62	C22 H28 O6 Na
	411.1749	1.8	4.4	20.5	91.0	8.73	C31 H23 O
	411.1808	-4.1	-10.0	11.5	89.7	32.66	C24 H27 O6

Figure S54. HR-ESI-MS spectrum of compound 7

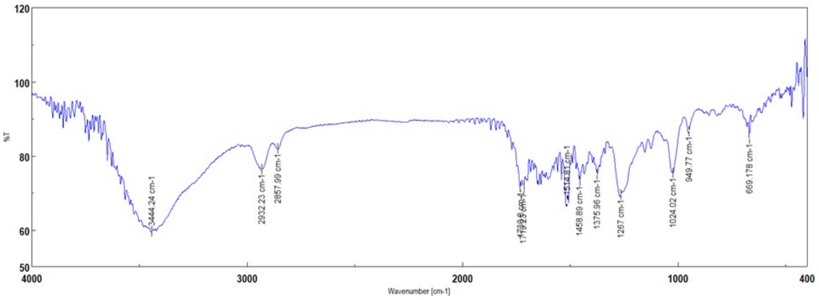

Figure S55. IR spectrum of compound 7

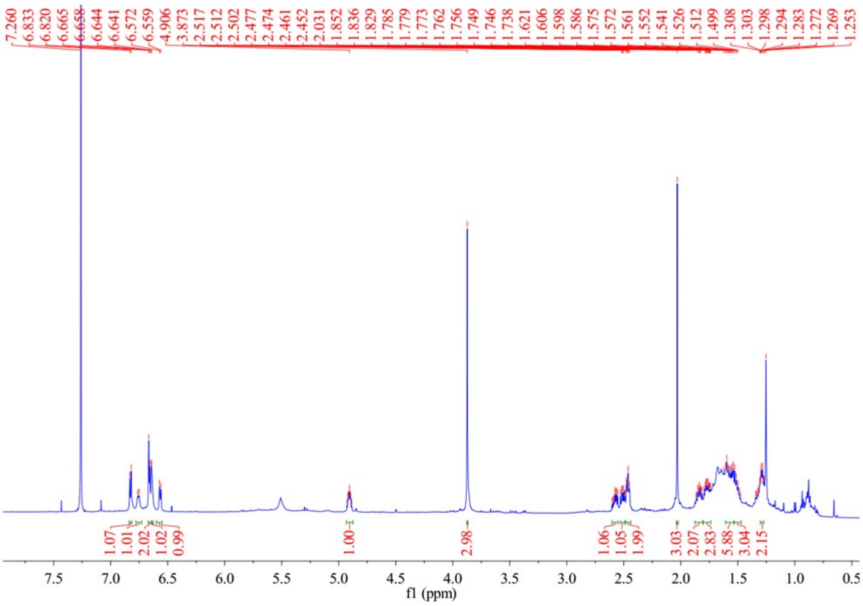

Figure S56. ^{1}H-NMR spectrum of compound 7

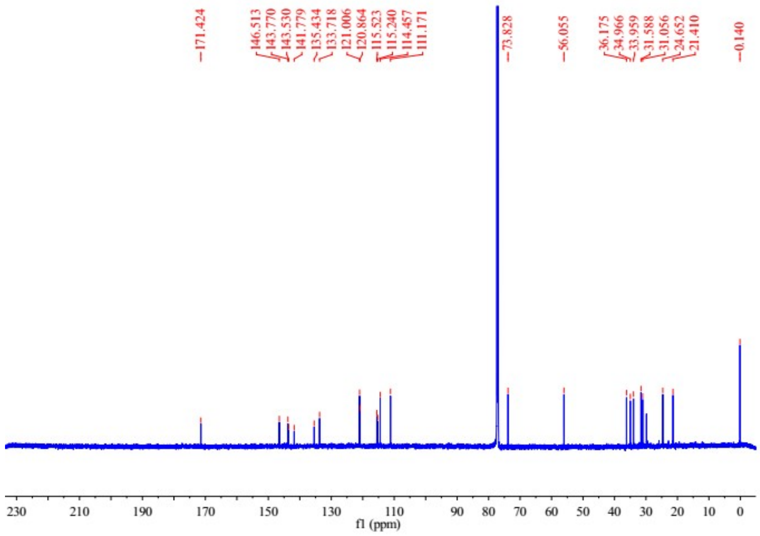

Figure S57. ^{13}C-NMR spectrum of compound 7

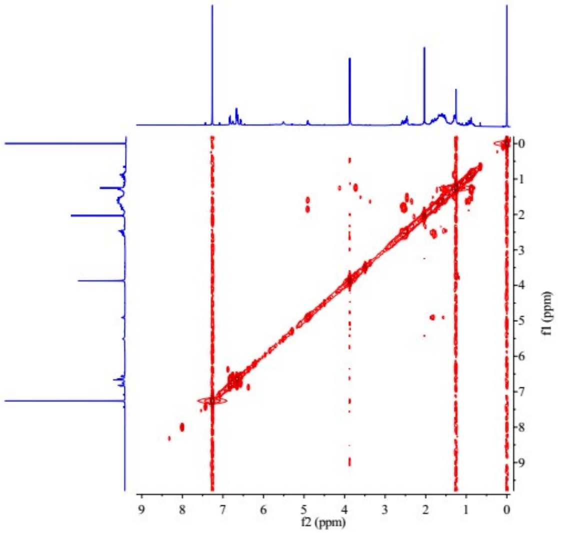

Figure S58. ^{1}H-^{1}H COSY spectrum of compound 7

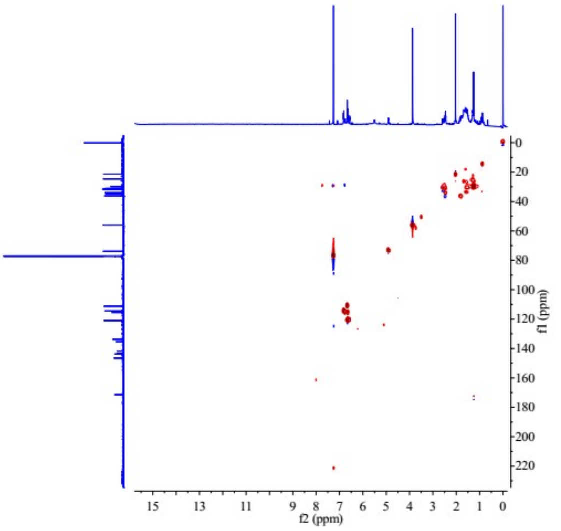

Figure S59. HSQC spectrum of compound 7

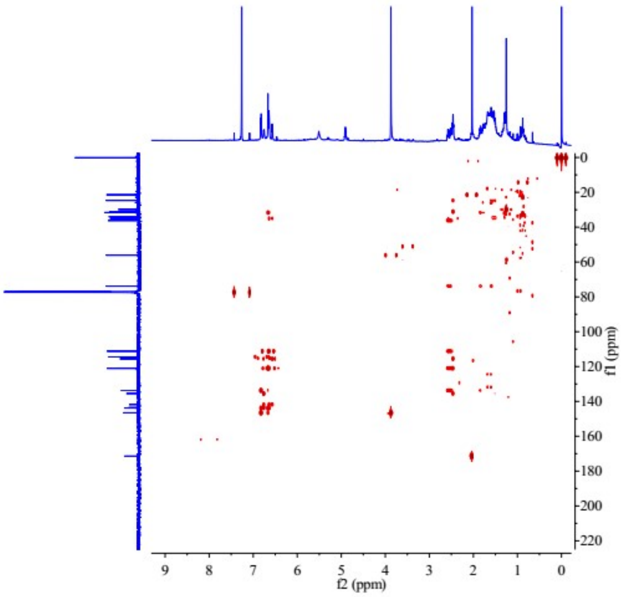

Figure S60. HMBC spectrum of compound 7

Compound 8

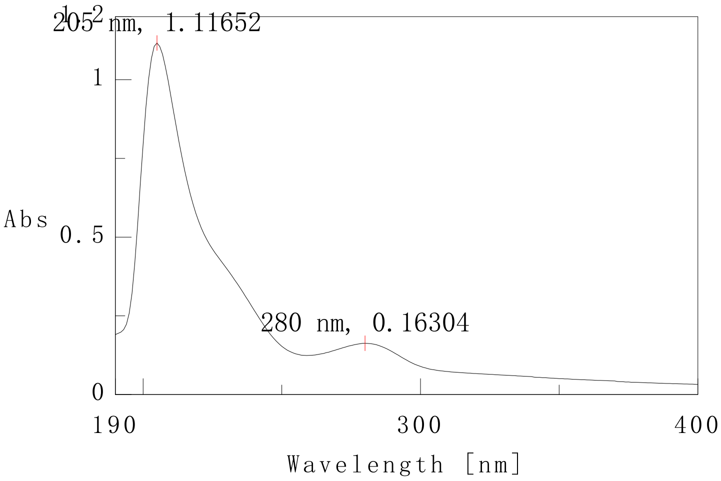

Figure S61. UV spectrum of compound 8

Elemental Composition Report — **Page 1**

Single Mass Analysis
Tolerance = 10.0 PPM / DBE: min = -1.5, max = 50.0
Element prediction: Off
Number of isotope peaks used for i-FIT = 3

Monoisotopic Mass, Even Electron Ions
190 formula(e) evaluated with 3 results within limits (all results (up to 1000) for each mass)
Elements Used:
C: 0-100 H: 0-500 O: 0-50 Na: 0-1

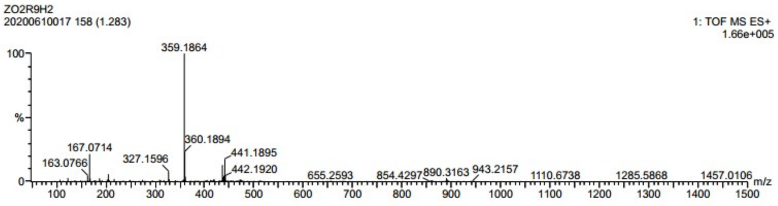

Minimum:				-1.5			
Maximum:		5.0	10.0	50.0			
Mass	Calc. Mass	mDa	PPM	DBE	i-FIT	Conf(%)	Formula
441.1895	441.1889	0.6	1.4	8.5	209.8	92.41	C23 H30 O7 Na
	441.1913	-1.8	-4.1	11.5	212.3	7.56	C25 H29 O7
	441.1855	4.0	9.1	20.5	217.8	0.03	C32 H25 O2

Figure S62. HR-ESI-MS spectrum of compound 8

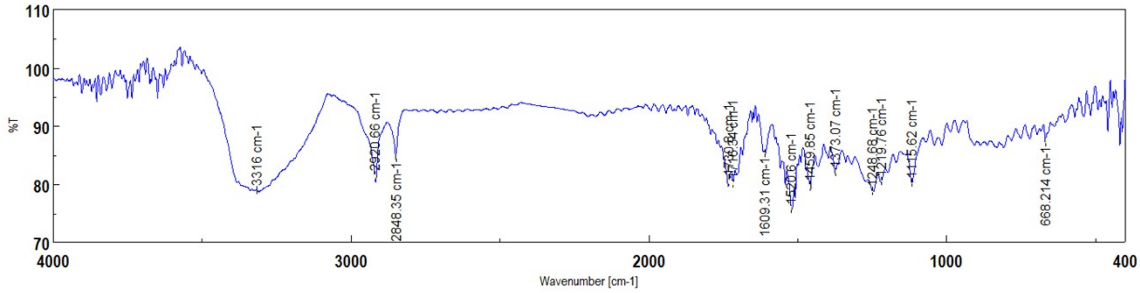

Figure S63. IR spectrum of compound 8

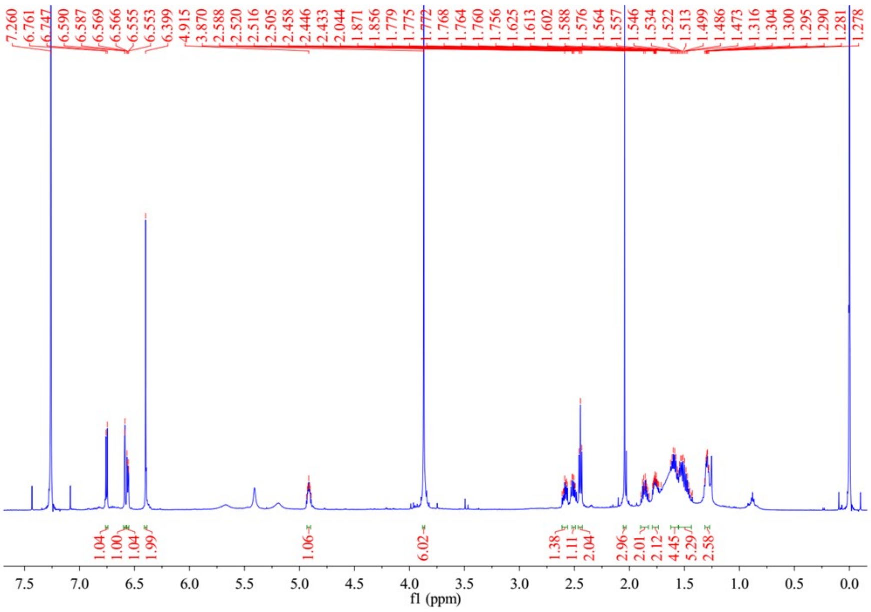

Figure S64. ^{1}H-NMR spectrum of compound 8

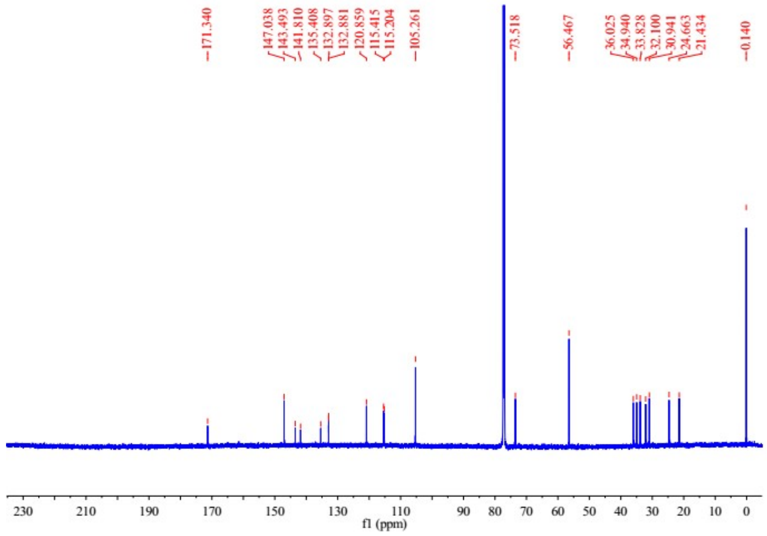

Figure S65. ^{13}C-NMR spectrum of compound 8

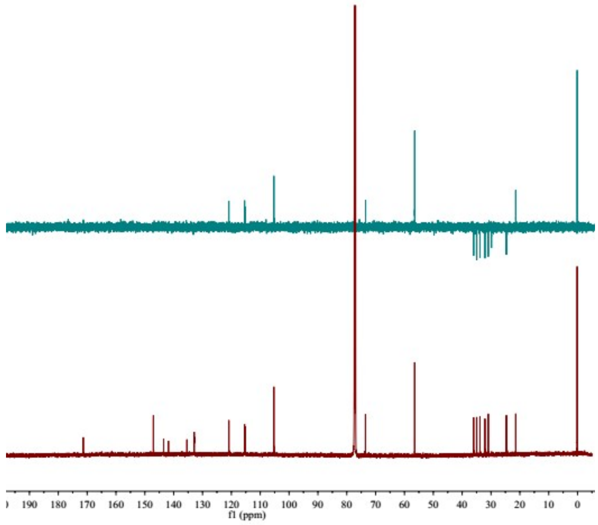

Figure S66. ^{13}**C-NMR and DEPT 135 spectra of compound 8**

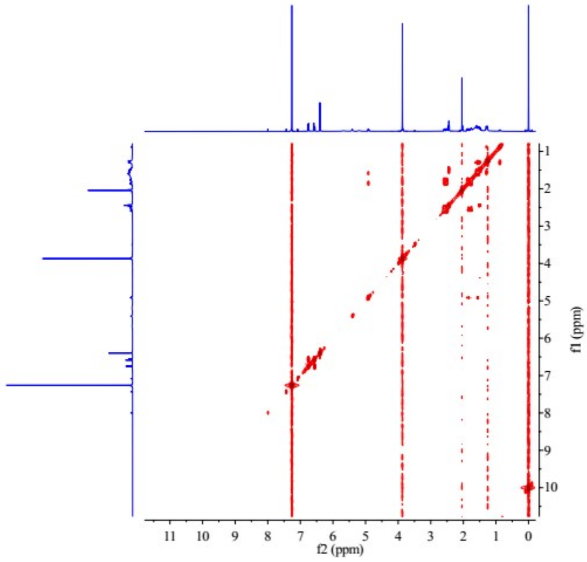

Figure S67. ^{1}**H-**^{1}**H COSY spectrum of compound 8**

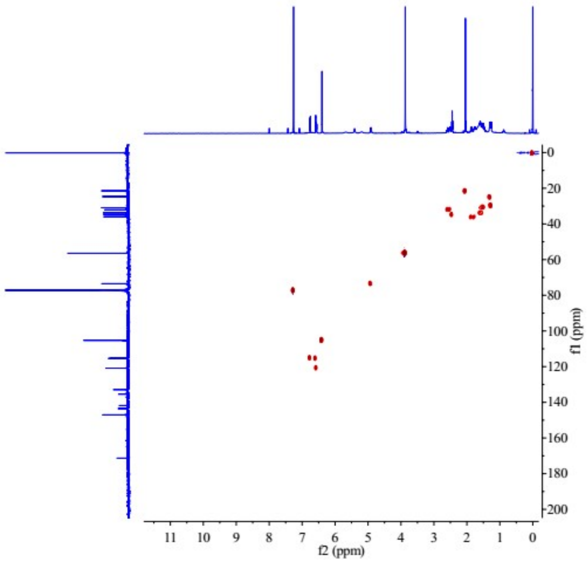

Figure S68. HSQC spectrum of compound 8

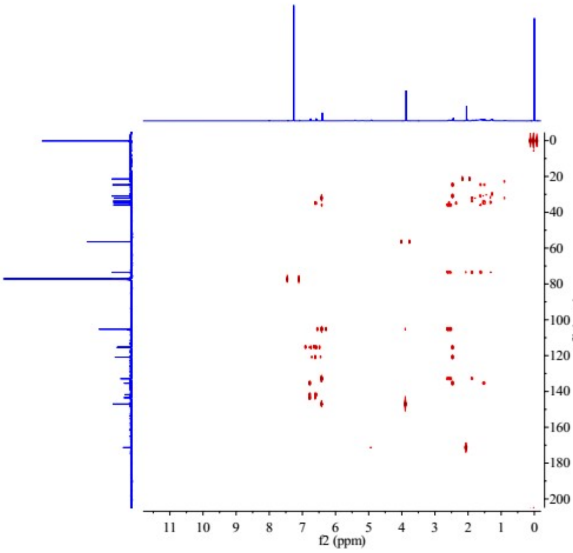

Figure S69. HMBC spectrum of compound 8

4. ECD calculation

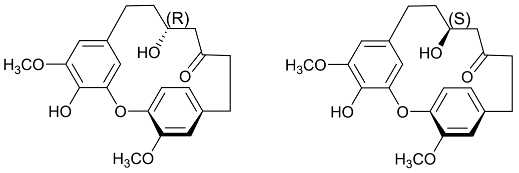

3a **3b**

Figure S70. The structures of compounds 3a and 3b.

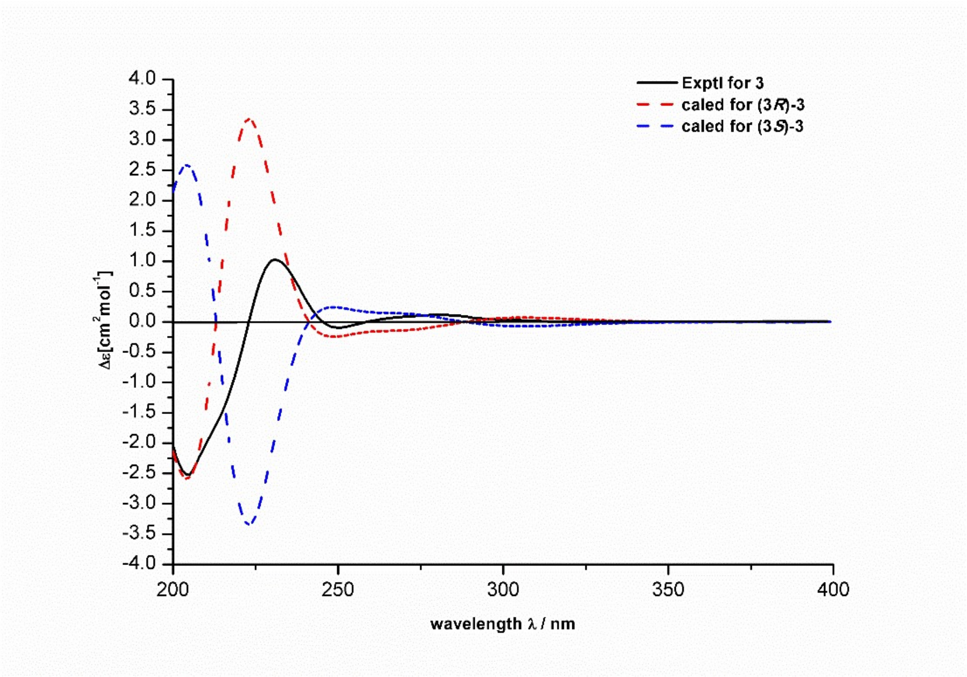

Figure S71. Experimental and calculated CD spectra of compound 3.

5. The western blotting assay results of A549 cell line

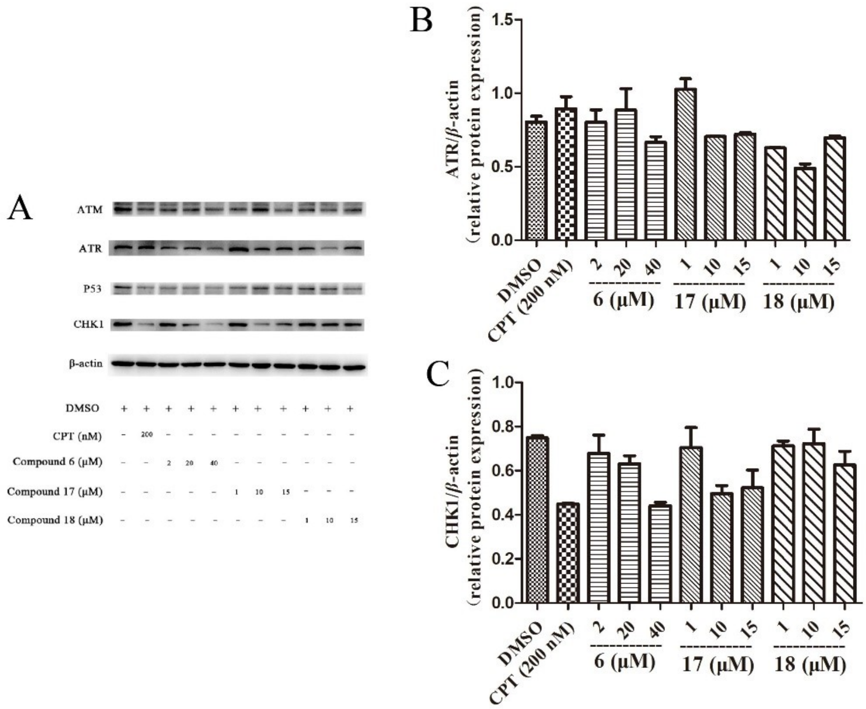

Figure S72. Effects of compounds **6**, **17**,and **18** on the protein expression of ATM, ATR, P53, and CHK1 in A549 cell line. A549 cells were pretreated with different concentrations of compounds **6**, **17**, and **18** for 24 h. The cells were lysed with RIPA buffer and the protein levels for total ATM, ATR, P53, and CHK1 were measured by using immunoblot analysis. *β*-Actin was used as a loading control. CPT was used as a positive control. And all of the experiments have been repeated three times independently.
