# Supplementary material for: Diarylheptanoid analogues from the rhizomes of Zingiber officinale and their anti-tumour activity
Source: RSC Adv. 2021 Sep 2;11(47):29376–84. doi: 10.1039/d1ra03592d (PMC9040573; doi:10.1039/d1ra03592d)
Supplement: RA-011-D1RA03592D-s002 [file RA-011-D1RA03592D-s002.pdf]

Electronic Supplementary Material (ESI) for RSC Advances.
This journal is © The Royal Society of Chemistry 2021

Western blots' raw data description

1. HCT116 Western blots data in the manuscript:

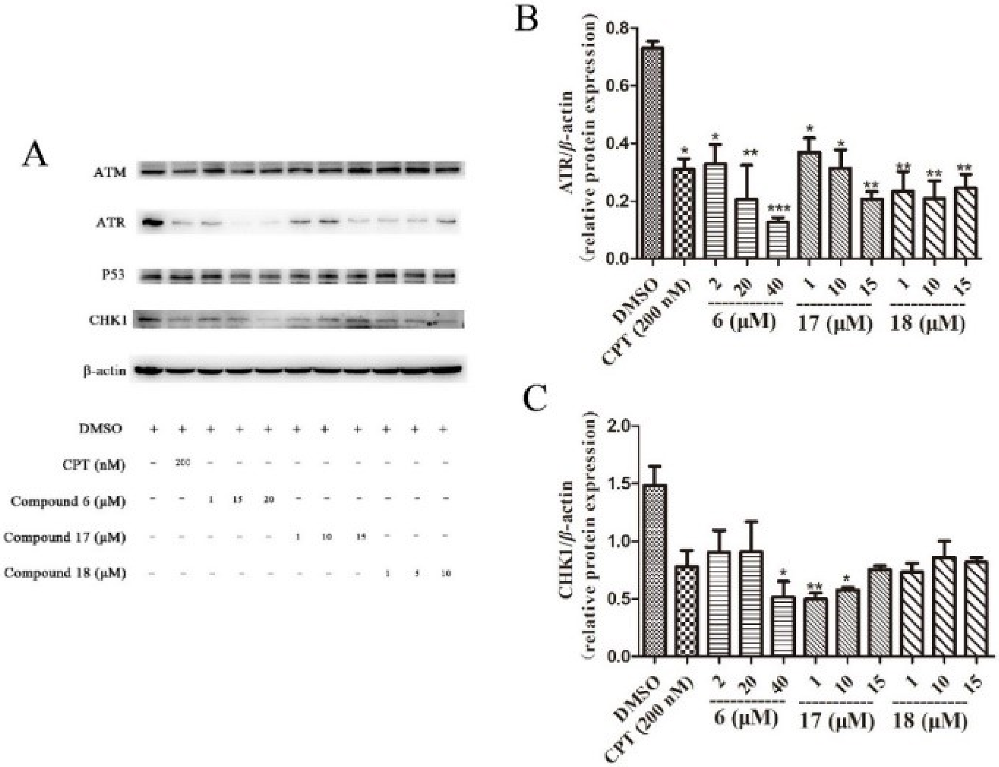

The corresponding raw data as following:

The first group

ATM:

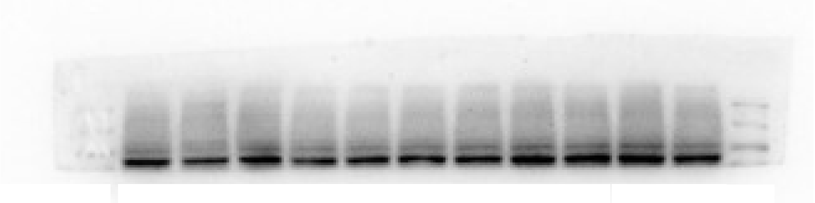

**Marker**	+	-	-	-	-	-	-	-	-	-	-	-	+
**DMSO**	-	+	+	+	+	+	+	+	+	+	+	+	-
**CPT (nM)**	-	-	200	-	-	-	-	-	-	-	-	-	-
**Comp 6 (μM)**	-	-	-	1	15	20	-	-	-	-	-	-	-
**Comp 17 (μM)**	-	-	-	-	-	-	1	10	15	-	-	-	-
**Comp 18 (μM)**	-	-	-	-	-	-	-	-	-	1	5	10	-

ATR:

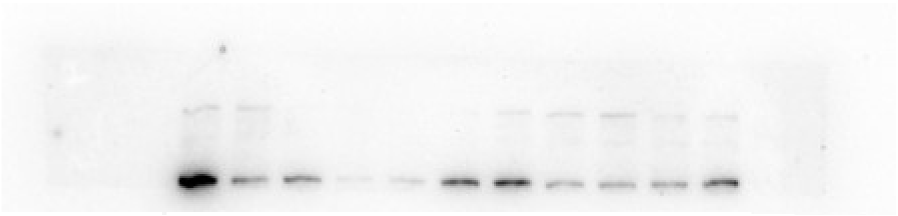

**Marker**	+	-	-	-	-	-	-	-	-	-	-	-	+
**DMSO**	-	+	+	+	+	+	+	+	+	+	+	+	-
**CPT (nM)**	-	-	200	-	-	-	-	-	-	-	-	-	-
**Comp 6 (μM)**	-	-	-	1	15	20	-	-	-	-	-	-	-
**Comp 17 (μM)**	-	-	-	-	-	-	1	10	15	-	-	-	-
**Comp 18 (μM)**	-	-	-	-	-	-	-	-	-	1	5	10	-

P53:

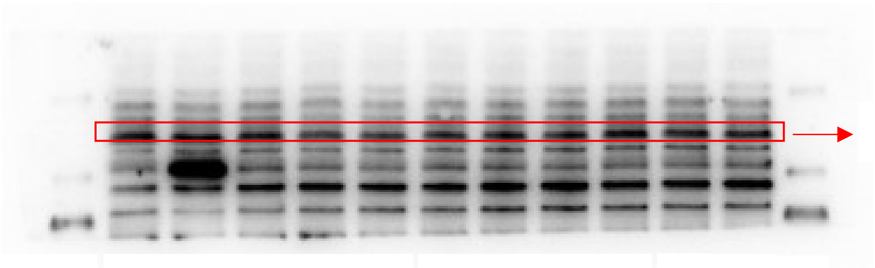

This protein band is P53.

**Marker**	+	-	-	-	-	-	-	-	-	-	-	-	+
**DMSO**	-	+	+	+	+	+	+	+	+	+	+	+	-
**CPT (nM)**	-	-	200	-	-	-	-	-	-	-	-	-	-
**Comp 6 (μM)**	-	-	-	1	15	20	-	-	-	-	-	-	-
**Comp 17 (μM)**	-	-	-	-	-	-	1	10	15	-	-	-	-
**Comp 18 (μM)**	-	-	-	-	-	-	-	-	-	1	5	10	-

CHK1:

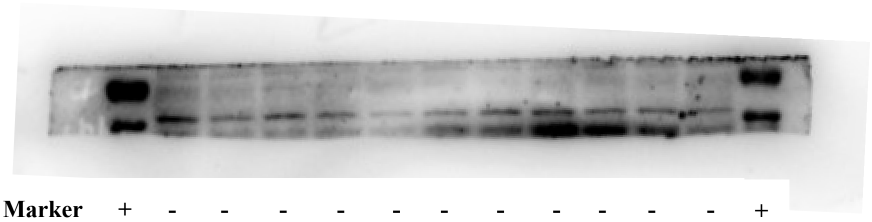

**Marker**	+	-	-	-	-	-	-	-	-	-	-	-	+
**DMSO**	-	+	+	+	+	+	+	+	+	+	+	+	-
**CPT (nM)**	-	-	200	-	-	-	-	-	-	-	-	-	-
**Comp 6 (μM)**	-	-	-	1	15	20	-	-	-	-	-	-	-
**Comp 17 (μM)**	-	-	-	-	-	-	1	10	15	-	-	-	-
**Comp 18 (μM)**	-	-	-	-	-	-	-	-	-	1	5	10	-

β-actin:

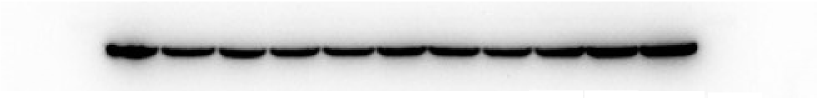

**Marker**	+	-	-	-	-	-	-	-	-	-	-	-	+
**DMSO**	-	+	+	+	+	+	+	+	+	+	+	+	-
**CPT (nM)**	-	-	200	-	-	-	-	-	-	-	-	-	-
**Comp 6 (μM)**	-	-	-	1	15	20	-	-	-	-	-	-	-
**Comp 17 (μM)**	-	-	-	-	-	-	1	10	15	-	-	-	-
**Comp 18 (μM)**	-	-	-	-	-	-	-	-	-	1	5	10	-

The second group:

ATM:

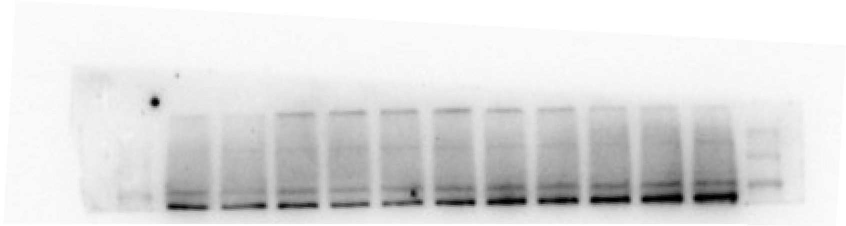

**Marker**	+	-	-	-	-	-	-	-	-	-	-	-	+
**DMSO**	-	+	+	+	+	+	+	+	+	+	+	+	-
**CPT (nM)**	-	-	200	-	-	-	-	-	-	-	-	-	-
**Comp 6 (μM)**	-	-	-	1	15	20	-	-	-	-	-	-	-
**Comp 17 (μM)**	-	-	-	-	-	-	1	10	15	-	-	-	-
**Comp 18 (μM)**	-	-	-	-	-	-	-	-	-	1	5	10	-

ATR:

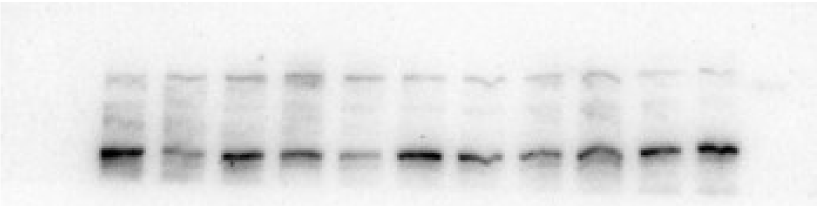

**Marker**	+	-	-	-	-	-	-	-	-	-	-	-	+
**DMSO**	-	+	+	+	+	+	+	+	+	+	+	+	-
**CPT (nM)**	-	-	200	-	-	-	-	-	-	-	-	-	-
**Comp 6 (μM)**	-	-	-	1	15	20	-	-	-	-	-	-	-
**Comp 17 (μM)**	-	-	-	-	-	-	1	10	15	-	-	-	-
**Comp 18 (μM)**	-	-	-	-	-	-	-	-	-	1	5	10	-

P53:

Marker DMSO CPT 6 6 6 17 17 17 18 18 18 Marker

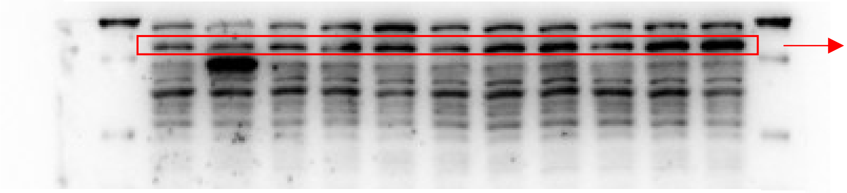

This protein band is P53.

**Marker**	+	-	-	-	-	-	-	-	-	-	-	-	+
**DMSO**	-	+	+	+	+	+	+	+	+	+	+	+	-
**CPT (nM)**	-	-	200	-	-	-	-	-	-	-	-	-	-
**Comp 6 (μM)**	-	-	-	1	15	20	-	-	-	-	-	-	-
**Comp 17 (μM)**	-	-	-	-	-	-	1	10	15	-	-	-	-
**Comp 18 (μM)**	-	-	-	-	-	-	-	-	-	1	5	10	-

CHK1:

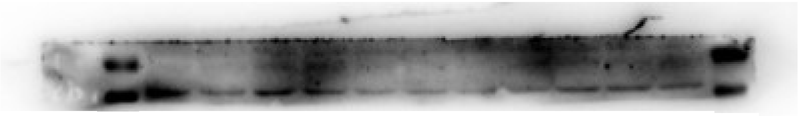

**Marker**	+	-	-	-	-	-	-	-	-	-	-	-	+
**DMSO**	-	+	+	+	+	+	+	+	+	+	+	+	-
**CPT (nM)**	-	-	200	-	-	-	-	-	-	-	-	-	-
**Comp 6 (μM)**	-	-	-	1	15	20	-	-	-	-	-	-	-
**Comp 17 (μM)**	-	-	-	-	-	-	1	10	15	-	-	-	-
**Comp 18 (μM)**	-	-	-	-	-	-	-	-	-	1	5	10	-

β-actin:

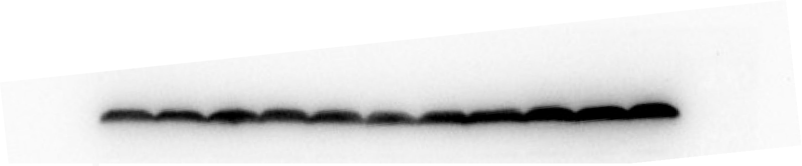

**Marker**	+	-	-	-	-	-	-	-	-	-	-	-	+
**DMSO**	-	+	+	+	+	+	+	+	+	+	+	+	-
**CPT (nM)**	-	-	200	-	-	-	-	-	-	-	-	-	-
**Comp 6 (μM)**	-	-	-	1	15	20	-	-	-	-	-	-	-
**Comp 17 (μM)**	-	-	-	-	-	-	1	10	15	-	-	-	-
**Comp 18 (μM)**	-	-	-	-	-	-	-	-	-	1	5	10	-

The third group:

ATM:

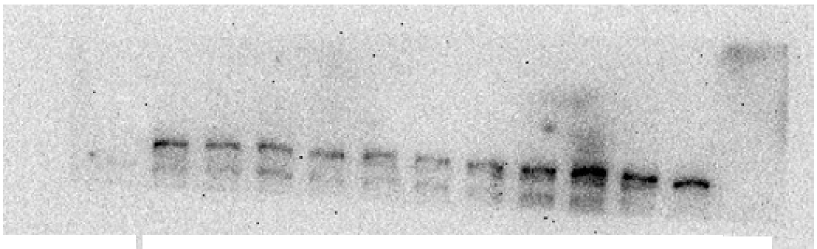

**Marker**	+	-	-	-	-	-	-	-	-	-	-	-	+
**DMSO**	-	+	+	+	+	+	+	+	+	+	+	+	-
**CPT (nM)**	-	-	200	-	-	-	-	-	-	-	-	-	-
**Comp 6 (μM)**	-	-	-	1	15	20	-	-	-	-	-	-	-
**Comp 17 (μM)**	-	-	-	-	-	-	1	10	15	-	-	-	-
**Comp 18 (μM)**	-	-	-	-	-	-	-	-	-	1	5	10	-

ATR:

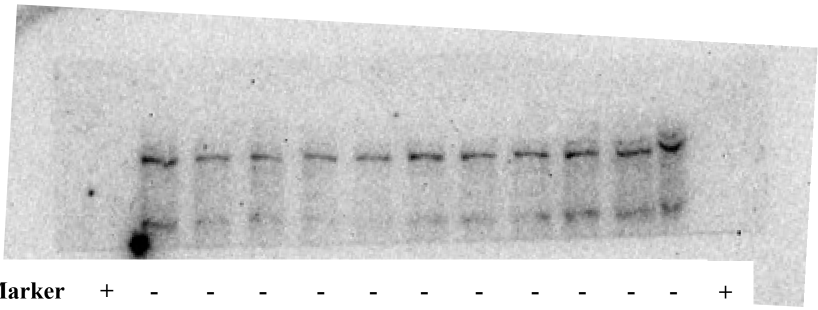

**Marker**	+	-	-	-	-	-	-	-	-	-	-	-	+
**DMSO**	-	+	+	+	+	+	+	+	+	+	+	+	-
**CPT (nM)**	-	-	200	-	-	-	-	-	-	-	-	-	-
**Comp 6 (μM)**	-	-	-	1	15	20	-	-	-	-	-	-	-
**Comp 17 (μM)**	-	-	-	-	-	-	1	10	15	-	-	-	-
**Comp 18 (μM)**	-	-	-	-	-	-	-	-	-	1	5	10	-

P53：

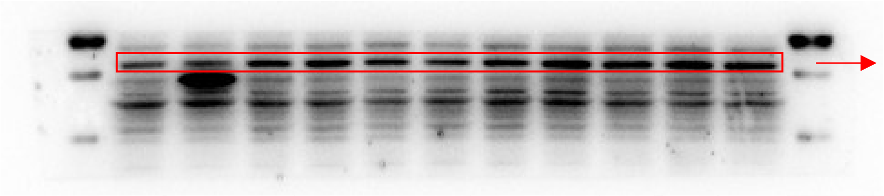

This protein band is P53.

**Marker**	+	-	-	-	-	-	-	-	-	-	-	-	+
**DMSO**	-	+	+	+	+	+	+	+	+	+	+	+	-
**CPT (nM)**	-	-	200	-	-	-	-	-	-	-	-	-	-
**Comp 6 (μM)**	-	-	-	1	15	20	-	-	-	-	-	-	-
**Comp 17 (μM)**	-	-	-	-	-	-	1	10	15	-	-	-	-
**Comp 18 (μM)**	-	-	-	-	-	-	-	-	-	1	5	10	-

CHK1：

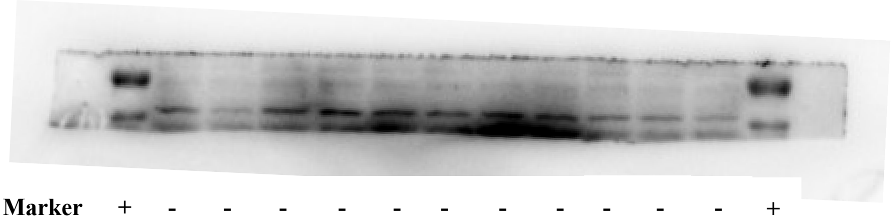

**Marker**	+	-	-	-	-	-	-	-	-	-	-	-	+
**DMSO**	-	+	+	+	+	+	+	+	+	+	+	+	-
**CPT (nM)**	-	-	200	-	-	-	-	-	-	-	-	-	-
**Comp 6 (μM)**	-	-	-	1	15	20	-	-	-	-	-	-	-
**Comp 17 (μM)**	-	-	-	-	-	-	1	10	15	-	-	-	-
**Comp 18 (μM)**	-	-	-	-	-	-	-	-	-	1	5	10	-

β-actin:

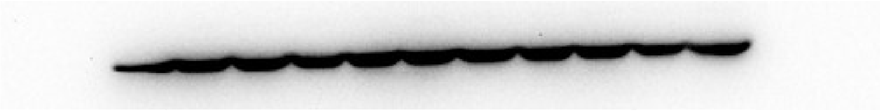

**Marker**	+	-	-	-	-	-	-	-	-	-	-	-	+
**DMSO**	-	+	+	+	+	+	+	+	+	+	+	+	-
**CPT (nM)**	-	-	200	-	-	-	-	-	-	-	-	-	-
**Comp 6 (μM)**	-	-	-	1	15	20	-	-	-	-	-	-	-
**Comp 17 (μM)**	-	-	-	-	-	-	1	10	15	-	-	-	-
**Comp 18 (μM)**	-	-	-	-	-	-	-	-	-	1	5	10	-

2. A549 Western blots data in the supple：

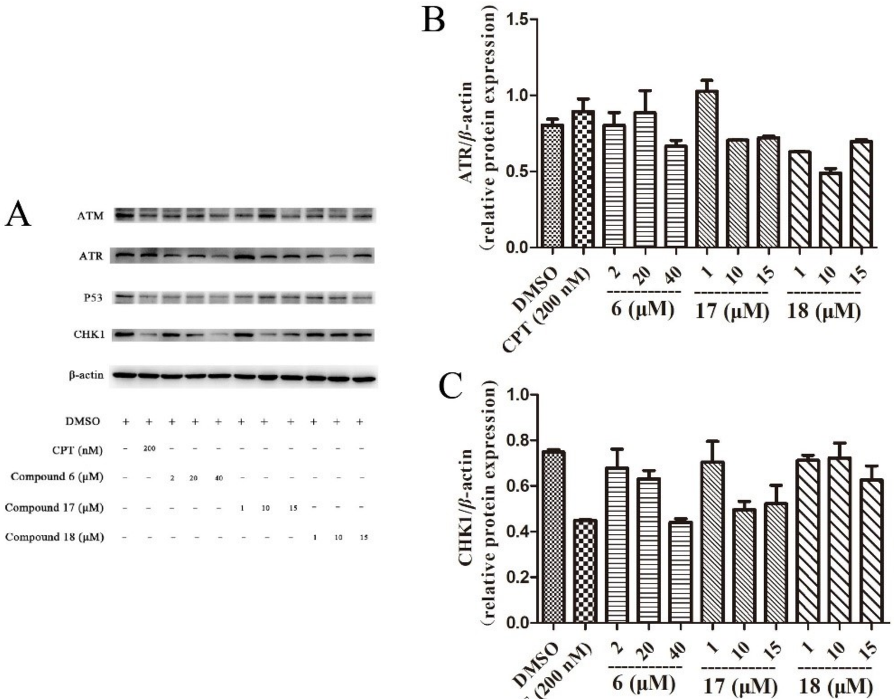

The corresponding raw data as following:

The first group:

ATM:

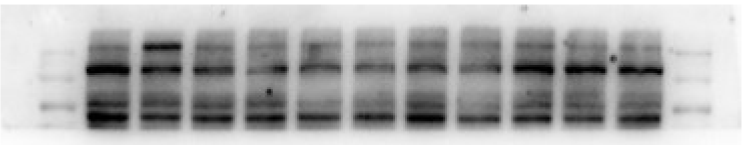

**Marker**	+	-	-	-	-	-	-	-	-	-	-	-	+
**DMSO**	-	+	+	+	+	+	+	+	+	+	+	+	-
**CPT (nM)**	-	-	200	-	-	-	-	-	-	-	-	-	-
**Comp 6 (μM)**	-	-	-	1	15	20	-	-	-	-	-	-	-
**Comp 17 (μM)**	-	-	-	-	-	-	1	10	15	-	-	-	-
**Comp 18 (μM)**	-	-	-	-	-	-	-	-	-	1	5	10	-

ATR:

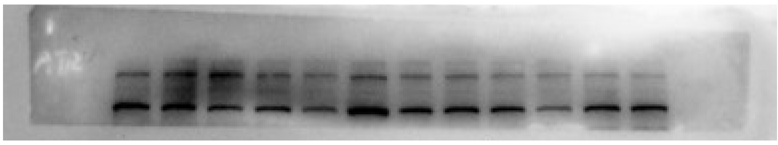

The molecular weight of ATR is similar to that of ATM. The protein was run separately under the same conditions.

**Marker**	+	-	-	-	-	-	-	-	-	-	-	-	-	+
**DMSO**	-	+	+	+	+	+	+	+	+	+	+	+	+	-
**CPT (nM)**	-	-	200	-	-	-	-	-	-	-	-	-	-	-
**Comp 6 (μM)**	-	-	-	1	15	20	-	-	-	-	-	-	-	-
**Comp 17 (μM)**	-	-	-	-	-	-	1	10	15	-	-	-	-	-
**Comp 18 (μM)**	-	-	-	-	-	-	-	-	-	1	5	10	10	-

P53:

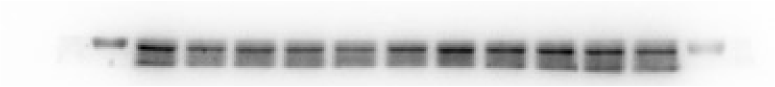

**Marker**	+	-	-	-	-	-	-	-	-	-	-	-	+
**DMSO**	-	+	+	+	+	+	+	+	+	+	+	+	-
**CPT (nM)**	-	-	200	-	-	-	-	-	-	-	-	-	-
**Comp 6 (μM)**	-	-	-	1	15	20	-	-	-	-	-	-	-
**Comp 17 (μM)**	-	-	-	-	-	-	1	10	15	-	-	-	-
**Comp 18 (μM)**	-	-	-	-	-	-	-	-	-	1	5	10	-

CHK1：

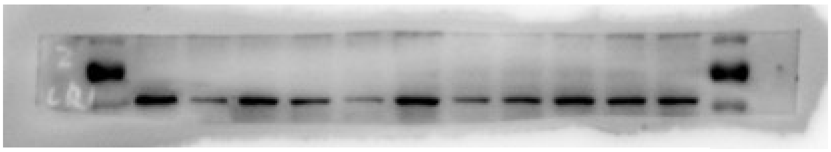

**Marker**	+	-	-	-	-	-	-	-	-	-	-	-	+
**DMSO**	-	+	+	+	+	+	+	+	+	+	+	+	-
**CPT (nM)**	-	-	**200**	-	-	-	-	-	-	-	-	-	-
**Comp 6 (μM)**	-	-	-	**1**	**15**	**20**	-	-	-	-	-	-	-
**Comp 17 (μM)**	-	-	-	-	-	-	**1**	**10**	**15**	-	-	-	-
**Comp 18 (μM)**	-	-	-	-	-	-	-	-	-	**1**	**5**	**10**	-

β-actin:

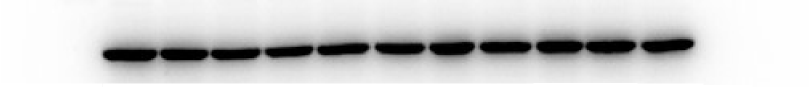

**Marker**	+	-	-	-	-	-	-	-	-	-	-	-	+
**DMSO**	-	+	+	+	+	+	+	+	+	+	+	+	-
**CPT (nM)**	-	-	**200**	-	-	-	-	-	-	-	-	-	-
**Comp 6 (μM)**	-	-	-	**1**	**15**	**20**	-	-	-	-	-	-	-
**Comp 17 (μM)**	-	-	-	-	-	-	**1**	**10**	**15**	-	-	-	-
**Comp 18 (μM)**	-	-	-	-	-	-	-	-	-	**1**	**5**	**10**	-

The second group:

ATM:

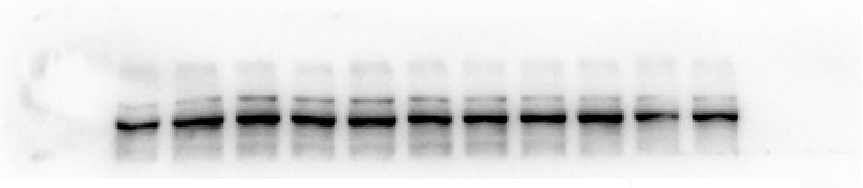

**Marker**	+	-	-	-	-	-	-	-	-	-	-	-	+
**DMSO**	-	+	+	+	+	+	+	+	+	+	+	+	-
**CPT (nM)**	-	-	**200**	-	-	-	-	-	-	-	-	-	-
**Comp 6 (μM)**	-	-	-	**1**	**15**	**20**	-	-	-	-	-	-	-
**Comp 17 (μM)**	-	-	-	-	-	-	**1**	**10**	**15**	-	-	-	-
**Comp 18 (μM)**	-	-	-	-	-	-	-	-	-	**1**	**5**	**10**	-

ATR:

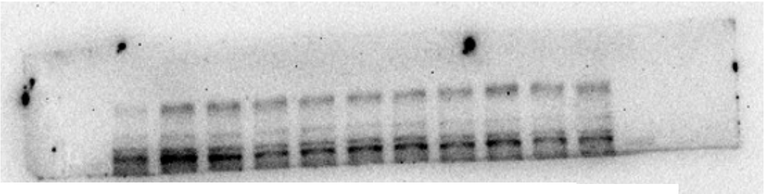

**Marker**	+	-	-	-	-	-	-	-	-	-	-	-	+
**DMSO**	-	+	+	+	+	+	+	+	+	+	+	+	-
**CPT (nM)**	-	-	200	-	-	-	-	-	-	-	-	-	-
**Comp 6 (μM)**	-	-	-	1	15	20	-	-	-	-	-	-	-
**Comp 17 (μM)**	-	-	-	-	-	-	1	10	15	-	-	-	-
**Comp 18 (μM)**	-	-	-	-	-	-	-	-	-	1	5	10	-

P53：

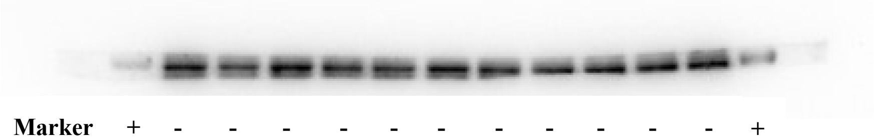

**Marker**	+	-	-	-	-	-	-	-	-	-	-	-	+
**DMSO**	-	+	+	+	+	+	+	+	+	+	+	+	-
**CPT (nM)**	-	-	200	-	-	-	-	-	-	-	-	-	-
**Comp 6 (μM)**	-	-	-	1	15	20	-	-	-	-	-	-	-
**Comp 17 (μM)**	-	-	-	-	-	-	1	10	15	-	-	-	-
**Comp 18 (μM)**	-	-	-	-	-	-	-	-	-	1	5	10	-

CHK1：

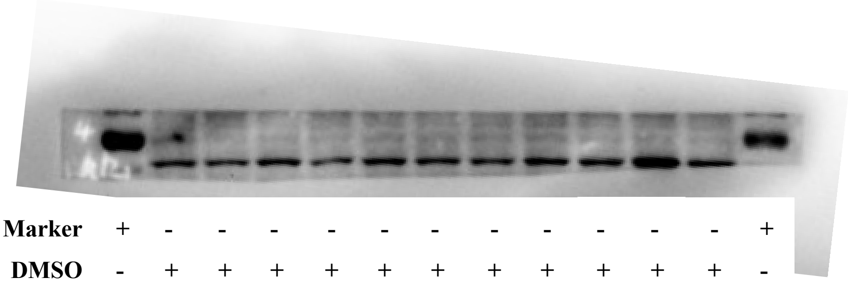

**Marker**	+	-	-	-	-	-	-	-	-	-	-	-	+
**DMSO**	-	+	+	+	+	+	+	+	+	+	+	+	-
**CPT (nM)**	-	-	200	-	-	-	-	-	-	-	-	-	-
**Comp 6 (μM)**	-	-	-	1	15	20	-	-	-	-	-	-	-
**Comp 17 (μM)**	-	-	-	-	-	-	1	10	15	-	-	-	-
**Comp 18 (μM)**	-	-	-	-	-	-	-	-	-	1	5	10	-

β-actin:

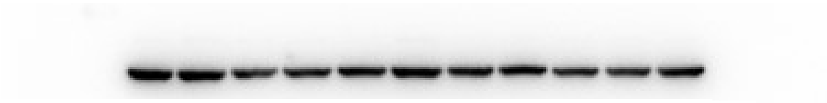

**Marker**	+	-	-	-	-	-	-	-	-	-	-	-	+
**DMSO**	-	+	+	+	+	+	+	+	+	+	+	+	-
**CPT (nM)**	-	-	**200**	-	-	-	-	-	-	-	-	-	-
**Comp 6 (μM)**	-	-	-	**1**	**15**	**20**	-	-	-	-	-	-	-
**Comp 17 (μM)**	-	-	-	-	-	-	**1**	**10**	**15**	-	-	-	-
**Comp 18 (μM)**	-	-	-	-	-	-	-	-	-	**1**	**5**	**10**	-

The third group:

ATM:

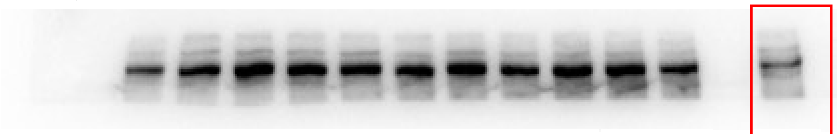

**Marker**	+	-	-	-	-	-	-	-	-	-	-	-	+	-
**DMSO**	-	+	+	+	+	+	+	+	+	+	+	+	-	+
**CPT (nM)**	-	-	**200**	-	-	-	-	-	-	-	-	-	-	-
**Comp 6 (μM)**	-	-	-	**1**	**15**	**20**	-	-	-	-	-	-	-	-
**Comp 17 (μM)**	-	-	-	-	-	-	**1**	**10**	**15**	-	-	-	-	-
**Comp 18 (μM)**	-	-	-	-	-	-	-	-	-	**1**	**5**	**10**	-	**10**

The molecular weight of ATR is similar to that of ATM. The protein was run separately under the same conditions. The last hole of the protein band is the band generated by the remaining sample after adding high doses of compound 18. The bands of CHK1 and P53 are [illegible]

ATR:

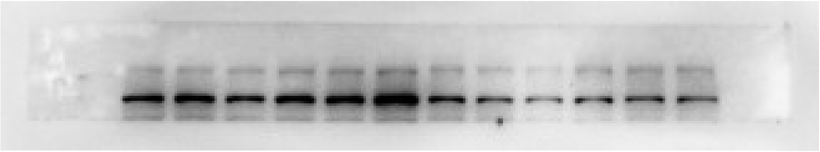

The molecular weight of ATR is similar to that of ATM. The protein was run separately under the same conditions.

**Marker**	+	-	-	-	-	-	-	-	-	-	-	-	-	+
**DMSO**	-	+	+	+	+	+	+	+	+	+	+	+	+	-
**CPT (nM)**	-	-	**200**	-	-	-	-	-	-	-	-	-	-	-
**Comp 6 (μM)**	-	-	-	**1**	**15**	**20**	-	-	-	-	-	-	-	-
**Comp 17 (μM)**	-	-	-	-	-	-	**1**	**10**	**15**	-	-	-	-	-
**Comp 18 (μM)**	-	-	-	-	-	-	-	-	-	**1**	**5**	**10**	**10**	-

P53:

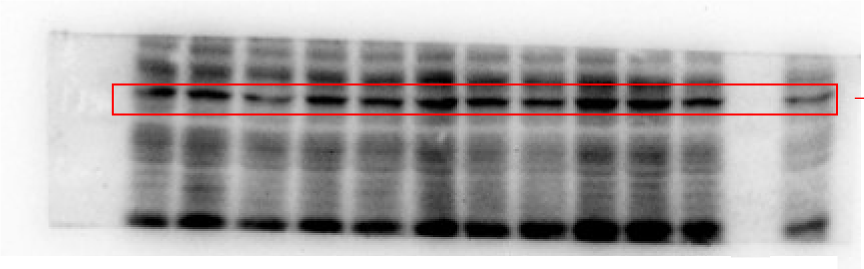

**Marker**	+	-	-	-	-	-	-	-	-	-	-	-	+	-
**DMSO**	-	+	+	+	+	+	+	+	+	+	+	+	-	+
**CPT (nM)**	-	-	200	-	-	-	-	-	-	-	-	-	-	-
**Comp 6 (μM)**	-	-	-	1	15	20	-	-	-	-	-	-	-	-
**Comp 17 (μM)**	-	-	-	-	-	-	1	10	15	-	-	-	-	-
**Comp 18 (μM)**	-	-	-	-	-	-	-	-	-	1	5	10	-	10

This protein band is P53.

CHK1:

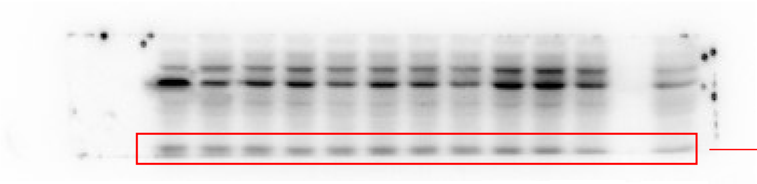

**Marker**	+	-	-	-	-	-	-	-	-	-	-	-	+	-
**DMSO**	-	+	+	+	+	+	+	+	+	+	+	+	-	+
**CPT (nM)**	-	-	200	-	-	-	-	-	-	-	-	-	-	-
**Comp 6 (μM)**	-	-	-	1	15	20	-	-	-	-	-	-	-	-
**Comp 17 (μM)**	-	-	-	-	-	-	1	10	15	-	-	-	-	-
**Comp 18 (μM)**	-	-	-	-	-	-	-	-	-	1	5	10	-	10

The protein band is a fragmented β-actin band that has been clipped, and this group of β-actin was repeated.

β-actin:

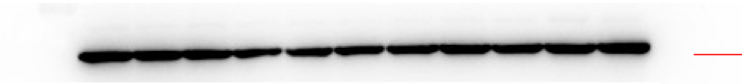

**Marker**	+	-	-	-	-	-	-	-	-	-	-	-	+
**DMSO**	-	+	+	+	+	+	+	+	+	+	+	+	-
**CPT (nM)**	-	-	200	-	-	-	-	-	-	-	-	-	-
**Comp 6 (μM)**	-	-	-	1	15	20	-	-	-	-	-	-	-
**Comp 17 (μM)**	-	-	-	-	-	-	1	10	15	-	-	-	-
**Comp 18 (μM)**	-	-	-	-	-	-	-	-	-	1	5	10	-

The β-actin band was cut into the CHK1 band, so β-actin was tested separately under the same conditions.
